# Translating medication effects for alcohol use disorder across preclinical, human laboratory, and clinical trial outcomes using meta-analysis

**DOI:** 10.1038/s41398-025-03473-6

**Published:** 2025-07-21

**Authors:** Steven J. Nieto, Suzanna Donato, Han Du, Lindsay R. Meredith, Wave-Ananda Baskerville, Kaitlin R. McManus, Molly Magill, Marcelo F. Lopez, Howard C. Becker, Lara A. Ray

**Affiliations:** 1 Department of Psychology, University of California at Los Angeles, Los Angeles, CA USA; 2 Department of Psychiatry and Behavioral Neurosciences, Wayne State University School of Medicine, Detroit, MI USA; 3 Center for Alcohol and Addiction Studies, Brown University School of Public Health, Providence, RI USA; 4 Department of Psychiatry and Behavioral Sciences, Medical University of South Carolina, Charleston, SC USA; 5 Department of Psychiatry and Biobehavioral Sciences, University of California at Los Angeles, Los Angeles, CA USA

**Keywords:** Clinical pharmacology, Addiction

## Abstract

Animal models are used for preliminary testing of novel compounds for alcohol use disorder (AUD). However, it is unclear whether early efficacy in preclinical models reliably predicts efficacy in human laboratory and clinical trials. We searched the literature for medications tested for AUD in preclinical models (i.e., two-bottle choice [2-BC] and operant reinstatement), human laboratory cue-reactivity, and randomized clinical trials (RCTs). For preclinical models, we computed medication effects on 2-BC alcohol preference and consumption (k = 77 studies, 14 medications) as well as operant reinstatement (k = 18 studies, 8 medications). For human laboratory studies, we computed medication effects on alcohol cue-induced craving (k = 36 studies, 15 medications). For RCTs, we computed medication effects on RCT endpoints including return to any drinking and return to heavy drinking (k = 139 studies, 19 medications). We used medication as the unit of analysis and applied the Williamson-York bivariate weighted least squares estimation to preserve the errors in both the independent and dependent variables. Medication effects on 2-BC alcohol preference ($\hat{\beta }$ = 0.04, *p* = 0.004) and reinstatement ($\hat{\beta }$ = 0.20, *p* = 0.05) were positively associated with medication effects on return to any drinking in human clinical trials but no associations were found on other RCT outcomes tested. Preclinical medications findings were not associated with medication effects on cue-induced craving in the human laboratory. Medication findings on 2-BC alcohol preference and operant reinstatement track medication effects on select clinical trial outcomes, specifically return to any drinking. This study provides empirical support for the association between medication effects across species and experimental models, a critical, yet untested, premise of preclinical studies in medications development.

## Introduction

Animal experimental models play an integral role in understanding the multidimensional aspects of alcohol use disorder (AUD) and inform the early stages of medication development for AUD. However, there is growing concern regarding the translational utility of animal experiments and their behavioral endpoints to clinical research on AUD [1]. The decline in psychiatric drug development programs has been partly attributed to the limited predictive validity of some animal models [2]. Nonetheless, experimental studies in animals allow for precise experimental control over several parameters that confound AUD research in humans, including genetics, the influence of environment (e.g., upbringing, stress), and previous experience with alcohol, drugs, and rewards in general. The use of experimental animals in research facilitates the examination of neurochemical, neurobiological, and neurophysiological factors that may ultimately translate to disease states in humans. These correlates can then facilitate the development of novel therapeutic targets for complex, heterogeneous disorders such as AUD. The primary objective of this study is to systematically test the translational utility of widely used animal models in predicting medication efficacy in the human laboratory and in randomized clinical trials (RCTs).

Potential medications for AUD are typically tested for initial efficacy in animal models using the two-bottle choice (2-BC) and operant reinstatement paradigms [3]. The 2-BC paradigm is frequently used to evaluate the rewarding properties of alcohol [4, 5] in the animal’s home-cage. This method entails a noninvasive, non-operant self-administration approach where animals are presented with the option to voluntarily consume alcohol or a non-alcohol beverage (typically water) orally, with either open or limited access [6]. The most prevalent iteration of this procedure is the chronic intermittent access schedule, wherein rodents are granted 24-h access to alcohol, three days per week, followed by repeated periods of deprivation (usually occurring every other day). Substantial evidence illustrates that intermittent access to alcohol progressively augments intake in rodents across various protocols [7–9], mirroring the transition from moderate, social drinking to excessive alcohol consumption often observed in individuals who drink heavily. The acute and chronic effects of compounds are often evaluated on quantity of alcohol consumption (grams/kilogram/day) and alcohol preference (ratio of alcohol consumed over total volume of water and alcohol consumed) during the 2-BC procedures.

Alcohol-associated stimuli can heighten alcohol craving, thereby triggering relapse to alcohol drinking in abstinent individuals with AUD, contributing to its chronic and relapsing nature [10, 11]. To mirror this phenomenon in animals, conditioning models have been developed. One such model is conditioned reinstatement, where the resumption of extinguished operant responding (e.g., lever pressing) is induced by noncontingent exposure to alcohol-related cues (i.e., cue lights) [12, 13]. Relative to 2-BC procedures, operant testing is more resource-intensive with greater costs in equipment and time. Operant paradigms require removing the animal from its home-cage and transporting them to an operant test chamber with its own inherent cues. The operant chamber results in many opportunities for the animal to form associations between environmental stimuli and the reinforcing value of alcohol. Reinstatement procedures are crucial for probing the neural mechanisms underlying craving and relapse [14], as well as for assessing the efficacy of pharmacological interventions in preventing alcohol craving and subsequent relapse analog in rodents.

Human laboratory studies, including alcohol cue-reactivity paradigms, are often conceptualized as a bridge between preclinical research and clinical trials [15]. These controlled settings allow for the quantification of proximal medication effects (e.g., subjective craving) under standardized conditions and are hypothesized to reflect the early clinical potential of AUD pharmacotherapies. To our knowledge, no studies have evaluated whether medications that show efficacy in preclinical models also reduce alcohol cue-induced craving in the human laboratory.

The choice of which animal paradigm to use to test potential medications for AUD is often subjective with no consensus on which animal models or paradigms should be prioritized. The hypothesis that medication effects on preclinical endpoints can predict human laboratory outcomes or alcohol drinking in RCTs remains untested. Recent studies have employed novel meta-analytic techniques to provide quantitative evidence in support of human behavioral pharmacology endpoints in predicting clinical trial outcomes. Ray, et al. [16] showed that medication effect sizes in alcohol challenge studies predict medication effect sizes in randomized clinical trials (RCTs). A similar approach was used to test the relationship between medication effect sizes on cue-induced craving in alcohol reactivity studies and medication effect sizes on abstinence and heavy drinking endpoints in RCTs. In contrast to alcohol challenge studies, alcohol cue-reactivity studies effect sizes were not statistically associated with medication effect sizes on abstinence and heavy drinking outcomes in RCTs [17]. Importantly, these novel meta-analytic methods can be applied to test whether medication effects on preclinical models used to study AUD are associated with efficacy in alcohol cue-reactivity human laboratory studies and RCTs. These findings will provide quantitative evidence regarding behavioral pharmacology methods, such as choice of paradigm, that can be prioritized to screen novel compounds for AUD at the preclinical level of analysis.

The aim of this study is to investigate whether medication effects observed in two-bottle choice and reinstatement studies are associated with medication effect sizes observed in the alcohol cue-reactivity paradigm and RCTs for AUD. We conducted an extensive literature search to identify medications examined using the 2-BC, operant reinstatement, alcohol cue-reactivity human laboratory studies, and in RCTs. Descriptive statistics from each study were used to compute medication effect sizes, and the Williamson-York regression method was used to assess the relationship between medication effect sizes in preclinical studies, cue-reactivity studies in the human laboratory, and those in RCTs. We hypothesized that medications that diminish alcohol consumption and preference in 2-BC or reduce reinstatement responding in preclinical models, compared to placebo, would be associated with reduced cue-induced craving in the human laboratory, and more favorable abstinence and drinking outcomes in RCTs. These findings will offer quantitative insights to guide the use of preclinical paradigms as an early screening tool for evaluating the efficacy of medications for AUD.

## Methods

The protocol for this meta-analytic project underwent scientific review at the National Institutes of Health (see NIH reporter Project Number: 5R21AA029771). The current study was not formally preregistered. The study codebook, data used for analyses, and analytic code, can be accessed via https://github.com/sbujarski. The funders/sponsors had no role in the current study and the authors declare no competing interests.

### Literature search strategy- preclinical studies

Inclusion criteria for preclinical studies were as follows: (1) empirical study including animal subjects, (2) two-bottle choice paradigm and/or self-administration with reinstatement paradigm, (3) paradigms include the administration of a pharmacological agent (either approved or being developed for the treatment of AUD) and a control condition, and (4) peer-reviewed article reported in the English language or translated into English. We cast a broad net when selecting 2-BC studies: any study that tested one of our target medications, measured consumption and/or preference with a 2-BC design (whether intermittent or continuous access), and reported sufficient descriptive statistics for effect-size calculation was included. We did not exclude studies based solely on factors such as intermittent vs. continuous schedules, presence vs. absence of deprivation periods, or the availability of more than two bottles; rather, we documented these methodological variations on coding sheets for future meta-analytic work. Similar to 2-BC preclinical studies, we cast a wide net when determining which operant reinstatement studies to include. Specifically, we incorporated any operant self-administration or reinstatement study that (1) tested one of our focal medications (i.e., medications that were also assessed in human laboratory or RCT settings), (2) provided sufficient descriptive statistics to calculate effect sizes, and (3) utilized a valid comparison group (placebo or vehicle). We did not exclude studies based on sex of the animals, vapor induction versus non-vapor induction, schedules of reinforcement, or other methodological details. In collaboration with the University of California, Los Angeles (UCLA) library and based on our prior RCT literature search [16, 18], a PubMed search was conducted to identify preclinical (i.e., animal) studies testing medications for AUD published through February 21, 2023. Individual searches were conducted for each of the 26 medications. These medications included: acamprosate, aripiprazole, baclofen, dutasteride, finasteride, gabapentin, ibudilast, idazoxan, indomethacin, isoflavone, ivermectin, mecamylamine, memantine, nalmefene, naloxone, naltrexone, olanzapine, ondansetron, paroxetine, prazosin, quetiapine, rimonabant, ritanserin, topiramate, varenicline, and zonisamide.

An example search string for naltrexone is provided as a reference: (((*“alcohol consumption”[Title/Abstract] OR “ethanol consumption”[Title/Abstract] OR “alcohol preference”[Title/Abstract] OR “ethanol preference”[Title/Abstract] OR “two-bottle choice”[Title/Abstract] OR “cue induced reinstatement”[Title/Abstract] OR “stress induced reinstatement”[Title/Abstract] OR “alcohol induced reinstatement”[Title/Abstract] OR “ethanol induced reinstatement”[Title/Abstract] OR “drug evaluation, preclinical/methods”[MeSH Terms] OR “conditioning, operant/drug effects”[MeSH Terms] OR “alcohol drinking/drug therapy”[MeSH Terms] OR “choice behavior/drug effects”[MeSH Terms]) AND “naltrexone”[Title/Abstract]) NOT (“review”[Publication Type] OR “systematic review”[Publication Type])) AND ((fft[Filter]) AND (animal[Filter]) AND (english[Filter]))*

Following the PubMed search, records were imported into a citation management system, Endnote X9 [19], for organization and removal of duplicate records. Studies that remained were then transferred to the systematic review platform JBI SUMARI [20] for further screening. Three trained independent raters (LM, WB, and SD) completed the abstract screening, with two preclinical experts (SN and ML) serving as consensus reviewers in cases of disagreement.

Full-text review and data extraction were carried out by two trained independent raters (SD and CA) and a consensus reviewer (SN and ML). Data extraction was informed by previous meta-analytic projects by the team [16, 17, 21] and in collaboration with preclinical experts (SN, ML, and HB) to ensure a comprehensive and informative review of each publication. In instances where data was not reported (e.g., means, standard error estimates), the software DigitizeIt [22] was used to extract the necessary information from published figures.

**Fig. 1:**
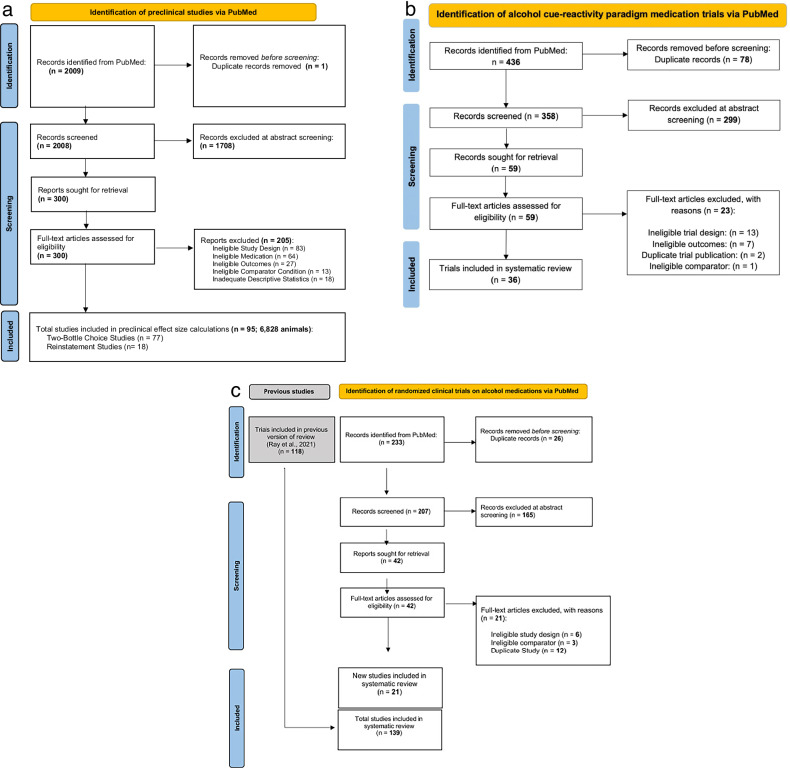
PRISMA flow diagram for systematic review of alcohol use disorder medication studies across different experimental paradigms. Preferred Reporting Items for Systematic reviews and Meta-Analyses (PRISMA) flow diagram of preclinical (**a**), cue-reactivity in the human laboratory (**b**), and randomized clinical trial records (**c**).

Preferred Reporting Items for Systematic Reviews and Meta-analyses (PRISMA) flow charts for this literature search are depicted in Fig. 1A. The literature search resulted in a total of 77 two-bottle choice studies across 14 medications and 18 reinstatement studies across 8 medications. A total of 6828 animals were included in these studies.

### Literature search strategy- cue-reactivity studies

A detailed description of the literature review method for cue-reactivity studies is reported in [23]. In short, a PubMed search was conducted for human laboratory studies published through January 3, 2022. Studies that met the following criteria were included in the meta-analysis: (1) randomized trial that included the administration of a pharmacological agent for the treatment of AUD (approved or being developed) along with a placebo comparison, (2) laboratory alcohol cue exposure paradigm (inclusive of neural alcohol cue-reactivity tasks), (3) cue-induced craving self-reports, and (4) peer-reviewed articles reported or translated into the English language.

The search resulted in a final sample of 36 cue-reactivity studies across 15 medications. There were a total of 1640 participants included in these studies. The PRISMA flow chart and risk of bias assessment was previously provided [23] but is also provided in Fig. 1B for convenience.

### Literature search strategy- randomized clinical trials

The literature search method for randomized clinical trials is detailed in [16, 18]. A PubMed search was conducted for randomized clinical trials on AUD medications published between January 2018 through April 2023. Inclusion criteria were as follows: (1) randomized trial, (2) double- or single-blinded, (3) placebo or active controlled, (4) primary endpoint includes any measure of alcohol consumption, (5) 4 or more weeks of treatment with the study medication, and (6) 12 or more weeks of follow-up after randomization. Results from this search were combined with a previous search of the literature (refer to [16]) to yield the final clinical trial dataset spanning from 1985–2023.

The search resulted in a final sample of 139 RCT studies across 19 medications that were included in the present meta-analysis. There were a total of 23,701 participants included in these RCT studies. The PRISMA flow chart for this literature search has been previously reported in [18] but is provided in Fig. 1C for convenience.

### Data analytic plan

#### Effect size estimation

We calculated unbiased Cohen’s *d* as the effect size for alcohol consumption and preference in each preclinical 2-BC study, alcohol lever presses in each preclinical reinstatement study, and cue-induced craving in each human laboratory cue-reactivity study. For all of these paradigms, Cohen’s *d* was defined as the difference between the mean of the active medication group and the mean of the control group, divided by the pooled standard deviation ($\frac{{\bar{y}}_{{medication}}-{\bar{y}}_{{control}}}{\sqrt{\frac{\left({n}_{{medication}}-1\right){s}_{{medication}}^{2}+\left({n}_{{control}}-1\right){s}_{{control}}^{2}}{{n}_{{medication}}+{n}_{{control}}-2}}}$ where ${n}_{{medication}}$ and ${n}_{{control}}$ are the sample sizes of the treatment and control groups, ${\bar{y}}_{{medication}}$ and ${\bar{y}}_{{control}}$ are the sample means of the treatment and control groups, and ${s}_{{medication}}^{2}$ and ${s}_{{control}}^{2}$ are the sample variances of the treatment and control groups). This effect size is commonly referred to as Hedges’ *g* [24] though Hedges noted its bias, especially with small per-study sample sizes [24, 25]. To address this bias, Hedges [24] proposed an unbiased correction to Hedges’ *g*, which was calculated using the formula $d=1-\frac{3}{4\left({n}_{{medication}}+{n}_{{control}}\right)-9}$. Negative values of Cohen’s *d* indicate lower alcohol consumption and preference (preclinical 2-BC studies), reduced lever presses (preclinical reinstatement studies), and lower cue-induced craving (human laboratory cue-reactivity studies) in the medication group compared to the control group.

For the RCTs, Cohen’s *d* was calculated for various outcomes related to drinking behavior (i.e., % Participants Return to Any Drinking, % Participants Return to Heavy Drinking, Percent Days Abstinent, Percent Heavy Drinking Days, Drinks per Day, and Drinks per Drinking Day). For most of these outcomes, Cohen’s *d* represented the difference between the means of the active medication group and the control group divided by the pooled standard deviation. An exception was the Percent Days Abstinent outcome, where Cohen’s *d* was calculated as the mean of the control group minus the mean of the active medication group divided by the pooled standard deviation to keep interpretation of the effect sizes consistent. Effect sizes were computed based on data from the active treatment periods across studies. In all RCT outcomes, negative effect sizes indicated greater effectiveness of the treatment group compared to the control group.

To calculate an average effect size per medication within each paradigm, we used fixed- and random-effects meta-analysis using the *metafor* R package [26]. Fixed-effects meta-analysis was used for reinstatement and cue-reactivity studies because there were too few studies to obtain an accurate estimate of the between studies heterogeneity. For 2-BC and RCTs, average effect sizes were derived for each medication and outcome, with between-study heterogeneity estimated using the DerSimonian-Laird (DL) estimator [27]. Heterogeneity was fixed at zero in cases where only one study was available. We did not assess strength of the evidence for preclinical, human laboratory, or RCT studies.

### Preclinical outcomes on cue-induced craving and RCT outcomes

To explore relationships between medication effect sizes in 2-BC, reinstatement, cue-reactivity studies and RCTs, we used Williamson-York bivariate weighted least squares estimation [28–31]. Statistical models were constructed to determine whether medication effect sizes on preclinical endpoints predict medication effect sizes on alcohol cue-reactivity in the human laboratory and medication effect sizes in RCTs.

We conducted one-tailed hypothesis tests to determine whether preclinical slopes were greater than zero (${H}_{a}:\beta\, >\, 0$: vs. ${H}_{0}:\beta =0$). Specifically, we used the Wald test to compare observed Z scores to the one-sided critical value of 1.64. We were unable to adjust for publication bias because the available correction methods rely on significant effect sizes and are primarily suited for fixed-effects meta-analysis.

## Results

### Medication effects on preclinical endpoints and cue-induced alcohol craving in humans

Williamson-York regression models were used to test the relationship between medication effects on preclinical endpoints (2-BC consumption and preference, lever presses in operant reinstatement studies) and medication effects on cue-induced alcohol craving in the human laboratory. For the preclinical 2-BC consumption endpoint, the linear slope is not statistically significant ($\hat{\beta }$ = 0.03, SE = 0.06, *p* = 0.30*)* for cue-induced craving (Supplemental Fig. 1A). For the preclinical 2-BC preference endpoint, the linear slope is not statistically significant ($\hat{\beta }$ = −0.12, SE = 0.05, *p* = 0.99*)* for cue-induced craving (Supplemental Fig. 1B). For the active lever presses endpoint within preclinical reinstatement studies, the linear slope is not statistically significant ($\hat{\beta }$ = 2.72, SE = 3.09, p = 0.18*)* for cue-induced craving (Supplemental Fig. 1C). Estimated effect sizes for medications used in 2-BC, operant reinstatement, and human laboratory cue-reactivity studies are presented in Supplemental Tables 1–3, respectively.

### Medication effects on preclinical endpoints and RCT outcomes

Williamson-York regression models were used to test the relationship between medication effects on preclinical endpoints (2-BC consumption and preference, and operant reinstatement) and medication effects on RCT endpoints including: % participants who return to any drinking, % participants who return to heavy drinking, percent days abstinent, percent days heavy drinking, drinks per day, and drinks per drinking day.

1. 2-BC, Operant Reinstatement, and Return to Any Drinking in RCTs
  For the preclinical 2-BC consumption endpoint, the linear slope is not statistically significant for return to any drinking in RCTs ($\hat{\beta }$ = −0.05, SE = 0.02, *p* = 0.98*;* Fig. 2A). For the preclinical 2-BC preference endpoint, the linear slope is statistically significant for return to any drinking in RCTs ($\hat{\beta }$ = 0.04, SE = 0.02, *p* = 0.004; Fig. 2B*)*, such that medications that reduced alcohol preference in preclinical 2-BC studies were associated with lower return to drinking in RCTs. For the preclinical active lever presses endpoint within reinstatement studies, the linear slope reached trend-level significance for return to heavy drinking in RCTs ($\hat{\beta }$ = 0.20, SE = 0.13, *p* = 0.05; Fig. 2C*)*, such that medications the reduced animal responding during reinstatement were associated with lower return to drinking in RCTs. Medication effect sizes on return to any drinking in RCTs are shown in Supplemental Table 4.

**Fig. 2:**
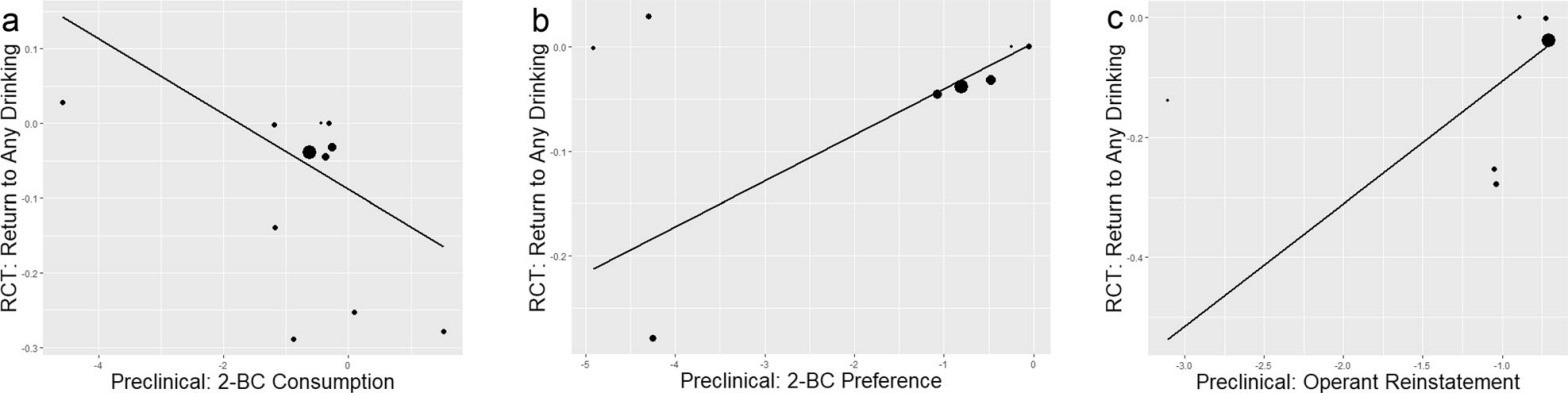
The linear relationship between medication effect sizes on preclinical endpoints and medication effect sizes on percent of participants who return to any drinking in randomized clinical trials. Williamson-York regression models with preclinical (2-BC alcohol consumption [**a**], 2-BC alcohol preference [**b**], and reinstatement [**c**]; x-axis) medication effect sizes predicting medication effect on the return to any drinking RCT endpoint; y-axis). Each medication appears as a dot on the regression line, with smaller dots indicating more error variance and larger dots indicating less error variance around each estimate. Negative effect sizes represent a favorable medication effect relative to placebo/active control.
2. 2-BC, Operant Reinstatement, and Return to Heavy Drinking in RCTs
  For the preclinical 2-BC consumption endpoint, the linear slope is not statistically significant for return to heavy drinking in RCTs ($\hat{\beta }$ = 0.01, SE = 0.04, *p* = 0.44*;* Fig. 3A). For the preclinical 2-BC preference endpoint, the linear slope is not statistically significant for return to heavy drinking in RCTs ($\hat{\beta }$ = 0.06, SE = 0.06, *p* = 0.17; Fig. 3B*)*. For the preclinical active lever presses endpoint within reinstatement studies, the linear slope is not statistically significant for return to heavy drinking in RCTs ($\hat{\beta }$ = 0.19, SE = 0.13, *p* = 0.08; Fig. 3C). Medication effect sizes on return to heavy drinking in RCTs are shown in Supplemental Table 4.

**Fig. 3:**
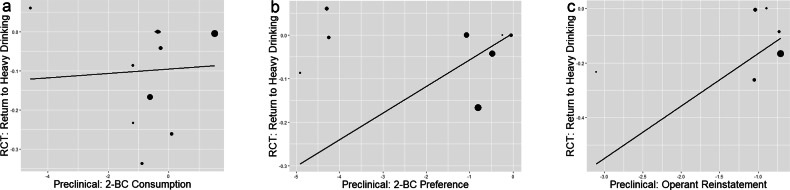
The linear relationship between medication effect sizes on preclinical endpoints and medication effect sizes on percent of participants who return to heavy drinking in randomized clinical trials. Williamson-York regression models with preclinical (2-BC alcohol consumption [**a**], 2-BC alcohol preference [**b**], and reinstatement [**c**]; x-axis) medication effect sizes predicting medication effect on the return to heavy drinking RCT endpoint; y-axis). Each medication appears as a dot on the regression line, with smaller dots indicating more error variance and larger dots indicating less error variance around each estimate. Negative effect sizes represent a favorable medication effect relative to placebo/active control.
3. 2-BC, Operant Reinstatement, and Percent Days Abstinent in RCTs
  For the 2-BC consumption endpoint, the linear slope is not statistically significant for percent days abstinent in RCTs ($\hat{\beta }$ = −0.05, SE = 0.03, *p* = 0.91*;* Supplemental Fig. 2A*)*. For the 2-BC preference endpoint, the linear slope is not statistically significant for percent days abstinent in RCTs ($\hat{\beta }$ = −0.06, SE = 0.05, *p* = 0.89; Supplemental Fig. 2B*)*. For the active lever presses endpoint within reinstatement studies, the linear slope is not statistically significant for percent days abstinent in RCTs ($\hat{\beta }$ = −0.08, SE = 0.06, *p* = 0.91; Supplemental Fig. 2C). Medication effect sizes on percent days abstinent in RCTs are shown in Supplemental Table 5.
4. 2-BC, Operant Reinstatement, and Percent Days Heavy Drinking in RCTs
  For the 2-BC consumption endpoint, the linear slope is not statistically significant for percent days heavy drinking in RCTs ($\hat{\beta }$ = 0.02, SE = 0.04, *p* = 0.32*;* Supplemental Fig. 3A*)*. For the 2-BC preference endpoint, the linear slope is not statistically significant for percent days heavy drinking in RCTs ($\hat{\beta }$ = −0.01, SE = 0.03, *p* = 0.62; Supplemental Fig. 3B). For the active lever presses endpoint within reinstatement studies, the linear slope is not statistically significant for percent days heavy drinking in RCTs ($\hat{\beta }$ = −0.05, SE = 0.03, *p* = 0.95; Supplemental Fig. 3C). Medication effect sizes on percent heavy drinking days in RCTs are shown in Supplemental Table 5.
5. 2-BC, Operant Reinstatement, and Drinks per Day in RCTs
  For the 2-BC consumption endpoint, the linear slope is not statistically significant for drinks per day in RCTs ($\hat{\beta }$ = 0.02, SE = 0.02, *p* = 0.11*;* Supplemental Fig. 4A*)*. For the 2-BC preference endpoint, the linear slope is not statistically significant for drinks per day in RCTs ($\hat{\beta }$ = −0.04, SE = 0.02, *p* = 0.96; Supplemental Fig. 4B*)*. For the active lever presses endpoint within reinstatement studies, the linear slope is not statistically significant for drinks per day in RCTs ($\hat{\beta }$ = −0.06, SE = 0.06, *p* = 0.87; Supplemental Fig. 4C). Medication effect sizes on drinks per day in RCTs are shown in Supplemental Table 6.
6. 2-BC, Operant Reinstatement, and Drinks per Drinking Day in RCTs

For the 2-BC consumption endpoint, the linear slope is not statistically significant for drinks per drinking day in RCTs ($\hat{\beta }$ = −0.05, SE = 0.04, *p* = 0.91; Supplemental Fig. 5A). For the 2-BC preference endpoint, the linear slope is not statistically significant for drinks per drinking day in RCTs ($\hat{\beta }$ = −0.09, SE = 0.04, *p* = 0.99; Supplemental Fig. 5B). For the active lever presses endpoint within reinstatement studies, the linear slope is not statistically significant for drinks per drinking day in RCTs ($\hat{\beta }$ = −0.02, SE = 0.09, *p* = 0.61; Supplemental Fig. 5C). Medication effect sizes on drinks per drinking day in RCTs are shown in Supplemental Table 6.

## Discussion

This study examined whether medication effects tested in widely used animal paradigms are associated with efficacy in human/clinical studies, namely cue-reactivity studies in the human laboratory and RCTs. Our laboratory has long been interested in the predictive value of animal and human laboratory models in relation to the RCT approach, the “gold standard” for medication development [3, 32]. This line of research is critical as the selection of preclinical paradigm to test a novel compound is largely subjective with little to no quantitative evidence available to prioritize animal paradigms in early efficacy testing of AUD medications. Leveraging previous systematic literature searches and using advanced meta-analytic methods, we found that favorable medication effects on both 2-BC preference and operant reinstatement were associated with favorable medication effects on a specific RCT endpoint (i.e., % participants who returned to any drinking). Preclinical 2-BC and reinstatement medication effect sizes were not associated with medication effects on other RCT outcomes, nor cue-induced alcohol craving in the human laboratory. To our knowledge, this is the first study to provide crucial evidence that medication effects on widely used preclinical models track medication effects in select RCT endpoints. These meta-analytic methods can be applied to future studies testing whether other preclinical models (i.e., progressive ratio self-administration) predict AUD medication effects in the human laboratory and RCTs.

The 2-BC and operant reinstatement are commonly used preclinical models to test novel compounds for AUD [33]. Our literature search of medication effects in 2-BC, which was guided by our previous medication search in RCTs [16, 18] (i.e., ensuring an RCT endpoint for meta-analysis testing), resulted in 77 studies across 14 medications and yielding 82 effect sizes for alcohol consumption and 38 effect sizes for alcohol preference. While most of the medications tested using the 2-BC paradigm significantly reduced both alcohol preference and consumption, there were some notable exceptions wherein acamprosate, baclofen, and ivermectin had nonsignificant effect sizes on 2-BC consumption. Memantine, ondansetron, and prazosin had nonsignificant effect sizes on 2-BC preference. It important to note that the small number of studies for certain medications (e.g., acamprosate in some 2-BC paradigms) can lead to wide confidence intervals or seemingly contradictory non-significant effects. Given that medications can have differing effects on endpoints within the same paradigm, evaluating several preclinical endpoints is needed to identify the preclinical endpoint(s) that predict clinical efficacy.

The degree to which preclinical outcomes predict AUD clinical trial outcomes is debated, yet mostly untested. Results from the current study showed that medication effects on alcohol preference within 2-BC studies were associated with favorable medication effects in RCTs. Specifically, we observed that medications that reduced alcohol preference in 2-BC studies also improved abstinence in RCTs via lower percentage of subjects that returned to any drinking. Medication effects on alcohol consumption in the 2-BC paradigm were not associated with medication effects on any RCT endpoint. This 2-BC finding is noteworthy, as a critical direction in using preclinical models in medication development relates to the identification and refinement of paradigms that have high external validity for predicting clinically relevant outcomes.

This study offers initial support for the translational validity of the 2-BC preference endpoint as it provides quantitative evidence that a medication’s effect on 2-BC preference at the preclinical level of analysis may be predictive of that medication’s success in Phase 2 clinical trials. In other words, the 2-BC preference outcome should be prioritized over 2-BC consumption in making go/no-go decisions early in the medication development pipeline. It is possible that 2-BC preference better isolates choice behavior under free-access conditions, potentially paralleling the real-world choice to “take or leave” alcohol. While the quantitative support is now provided it is important to acknowledge the limited number of medications with both 2-BC preference and RCT data. This recommendation should be revised as more studies become available for an updated meta-analysis.

Based on previous literature searches from our laboratory [16, 18, 23], we identified 18 operant reinstatement studies that tested AUD medications that were also tested in cue-reactivity and RCT studies. From these 18 studies, we calculated 21 effect sizes across 8 medications. Almost all of the identified medications were significantly effective at reducing operant reinstatement compared to placebo, with the exception of ivermectin. In support of our hypothesis, we found that medication effects on reinstatement were associated with medication effects on RCT outcomes. The positive slope for reinstatement on the % participants return to any drinking RCT outcome trended towards statistical significance, suggesting an initial signal whereby medications that reduce active lever presses during reinstatement are effective in improving abstinence in RCTs. While the meta-analytic approach used in the current study allowed us to leverage multiple effect sizes across medications, we may have been underpowered to detect statistical significance, particularly for reinstatement, as medication was the unit of analysis in the regression models and the analyses were limited to only those medications that were evaluated for efficacy in an animal model and at the human laboratory or clinical level. Therefore, considering the directionality of the effects in addition to their statistical significance is warranted. It is plausible that statistical power for these models can be improved as more medications and studies are added to the database, a future endeavor that will be labor- and resource-intensive but may have tremendous benefit for AUD medication development.

While the average effect of preclinical 2-BC and reinstatement medication effect sizes did not robustly predict efficacy on cue-induced craving in the human laboratory, the translational utility of human laboratory paradigms themselves has also been debated with little to no explicit evidence that medication findings in the cue-reactivity paradigm predict clinical efficacy on clinical trial outcomes [17]. Reinstatement in rodents may map more onto actual relapse-like behavior, whereas cue-induced craving in humans is a narrower measure of subjective desire that may not always predict drinking outcomes. This further underscores the complexity of craving as a self-reported measure in humans compared to a direct behavioral measure of seeking in rodents. Future work can examine the correlation between medication effects on preclinical endpoints and subjective response to alcohol in human laboratory studies, the latter of which has shown to predict medication effects in clinical trials [16]. It is important to note that there were a number of differences across experiments in preclinical studies. While the use of random-effects meta-analysis accounts for the heterogeneity between studies, future work can leverage meta-regression to examine the influence of study-level factors (i.e., species, strain, sex, drinking history, housing conditions) on the strength of observed medication effect sizes in preclinical studies. Future meta-analyses would benefit from (1) new vapor-chamber studies testing the same medications used in clinical trials, and (2) a broader set of endpoints (for instance, more direct measures of withdrawal or dependence severity) to explore potential moderators of medication efficacy in physically dependent animals. As these data become available, integrating them into an expanded dataset could yield deeper insights into how robustly medication signals generalize across the entire spectrum of AUD severity from intermittent heavy drinkers to individuals with marked dependence phenotypes. These analyses are beyond the scope of the current study but such information can help refine preclinical models to better capture a medication signal.

The current findings should be interpreted in light of the study’s strengths and limitations. Strengths include a robust sample of medication effect sizes collected across stages of medication development, meta-analytic methods that account for between study heterogeneity, and advanced regression models that consider errors in both the independent and dependent variables. A notable limitation of the study is the low sample size (medication was the unit of analysis) and potential for type 2 error as the regression models only included medications that were studied in 2-BC or reinstatement studies and cue-reactivity studies or RCTs. There are a host of medications that could not be included in the study; including many compounds tested in preclinical models that never proceeded to clinical testing [32, 34]. Effect-size estimates may be inflated for preclinical studies due to the absence of a placebo effect. While the current study used random-effects meta-analytic models, which incorporate between-study variance, we did not apply an explicit correction factor for potential overestimation in animal work. We emphasize this point as a caveat for interpretation and encourage future research to develop standardized adjustments. Additionally, this study cannot speak to other preclinical endpoints (i.e., progressive ratio self-administration) that are often used as early efficacy outcomes in medication development for AUD.

Overall, our findings show that medication effects on both alcohol preference in 2-BC studies and operant reinstatement track medication effects on return to any drinking in RCTs. These results highlight that testing medications on the 2-BC preference endpoint and operant reinstatement may maximize a medication signal if one exists. This study provides empirical support for the association between medication effects across species and experimental models, a critical, yet untested, premise of preclinical studies in medications development. In addition, this is the first study to provide quantitative data to help guide the decision of which preclinical endpoint to use as an early efficacy signal in AUD medication development. Future work can apply the methods used in the current study to test the utility of other preclinical models in predicting medication effects in the human laboratory and in RCTs.

## Supplementary information

Supplemental Tables and Figures
