# Supplementary material for: Translating medication effects for alcohol use disorder across preclinical, human laboratory, and clinical trial outcomes using meta-analysis
Source: Transl Psychiatry. 2025 Jul 21;15:250. doi: 10.1038/s41398-025-03473-6 (PMC12279940; doi:10.1038/s41398-025-03473-6)
Supplement: Supplementary file 1 — Supplemental Tables and Figures [file 41398_2025_3473_MOESM1_ESM.docx]

**Supplemental Table 1.** Estimated effect sizes and standard errors for medications tested in preclinical two-bottle (2-BC) choice studies

| **Medication** | **Number of Consumption Effect Sizes** | **Cohen’s *d* (**$\boldsymbol{\pm}$**SE)**  **for Consumption** | **Number of Preference Effect Sizes** | **Cohen’s *d* (**$\boldsymbol{\pm}$**SE)**  **for Preference** |
| --- | --- | --- | --- | --- |
| Acamprosate | 9 | 1.53 (2.66) | 7 | **-4.25 (2.26)** |
| Baclofen | 8 | 0.10 (0.39) |  |  |
| Gabapentin | 1 | **-0.89 (0.47)** |  |  |
| Ibudilast | 1 | **-3.62 (0.81)** |  |  |
| Ivermectin | 2 | -3.00 (3.21) | 2 | **-2.99 (2.79)** |
| Levetiracetam | 1 | **-4.58 (1.02)** | 1 | **-4.29 (0.97)** |
| Memantine | 2 | **-0.44 (0.28)** | 1 | -0.25 (0.37) |
| Naltrexone | 30 | **-0.63 (0.10)** | 12 | **-0.81 (0.65)** |
| Ondansetron | 3 | **-0.31 (0.21)** | 2 | -0.05 (0.31) |
| Prazosin | 6 | **-0.74 (0.19)** | 2 | -0.51 (1.01) |
| Rimonabant | 1 | **-1.18 (0.57)** |  |  |
| Ritanserin | 3 | **-0.36 (0.26)** | 5 | **-1.07 (0.67)** |
| Topiramate | 6 | **-0.26 (0.13)** | 3 | **-0.48 (0.23)** |
| Varenicline | 9 | **-1.19 (0.67)** | 3 | **-4.91 (4.24)** |

*Note*. Negative effect sizes indicate a more favorable medication effect. Bold indicates significant medication effect sizes.

**Supplemental Table 2.** Estimated effect sizes and standard errors for medications tested in preclinical operant reinstatement studies

| **Medication** | **Number of Reinstatement Effect Sizes** | **Cohen’s *d* (**$\boldsymbol{\pm}$**SE) for Reinstatement** |
| --- | --- | --- |
| Acamprosate | 1 | **-1.04 (0.52)** |
| Baclofen | 2 | **-1.05 (0.31)** |
| Ivermectin | 1 | -0.30 (0.38) |
| Naltrexone | 10 | **-0.70 (0.16)** |
| Ondansetron | 1 | **-0.89 (0.47)** |
| Prazosin | 3 | **-0.59 (0.26)** |
| Rimonabant | 1 | **-3.11 (0.66)** |
| Varenicline | 2 | **-0.72 (0.33)** |

*Note*. Negative effect sizes indicate a more favorable medication effect. Bold indicates significant medication effect sizes.

**Supplemental Table 3.** Estimated effect sizes and standard errors for medications tested on cue-induced craving in the human laboratory

| **Medication** | **Number of Cue-induced Craving Effect Sizes** | **Cohen’s *d* (**$\boldsymbol{\pm}$**SE) for Cue-induced Craving** |
| --- | --- | --- |
| Acamprosate | 4 | 0.05 (0.14) |
| Baclofen | 10 | **-0.17 (0.13)** |
| Ibudilast | 1 | -0.05 (0.29) |
| Ivermectin | 1 | 0.34 (0.43) |
| Memantine | 2 | **-0.45 (0.16)** |
| Nalmefene | 3 | **-0.25 (0.19)** |
| Naltrexone | 12 | **-0.24 (0.08)** |
| Olanzapine | 3 | **-0.36 (0.19)** |
| Ondansetron | 2 | -0.02 (0.29) |
| Prazosin | 30 | **-0.29 (0.27)** |
| Quetiapine | 1 | 0.18 (0.54) |
| Varenicline | 3 | 0.14 (0.15) |

*Note*. Negative effect sizes indicate a more favorable medication effect. Bold indicates significant medication effect sizes.

**Supplemental Table 4.** Estimated effect sizes and standard errors for medications tested on return to any drinking and return to heavy drinking in randomized clinical trials

| **Medication** | **Number of Return to Any Drinking Effect Sizes** | **Cohen’s *d* (**$\boldsymbol{\pm}$**SE) for Return to Any Drinking** | **Number of Return to Heavy Drinking Effect Sizes** | **Cohen’s *d* (**$\boldsymbol{\pm}$**SE) for Return to Heavy Drinking** |
| --- | --- | --- | --- | --- |
| Acamprosate | 28 | **-0.28 (0.05)** | 28 | 0.00 (0.03) |
| Aripiprazole | 1 | 0.45 (0.18) | 1 | 0.00 (0.15) |
| Baclofen | 13 | **-0.25 (0.12)** | 11 | **-0.26 (0.09)** |
| Carbamazepine | 1 | **-0.52 (0.45)** | 1 | 0.49 (0.51) |
| Gabapentin | 8 | **-0.29 (0.14)** | 8 | **-0.34 (0.10)** |
| Levetiracetam | 3 | 0.03 (0.13) | 3 | 0.06 (0.11) |
| Memantine | 1 | 0.00 (0.34) | 1 | 0.00 (0.34) |
| Nalmefene | 7 | 0.00 (0.05) | 7 | -0.03 (0.05) |
| Naltrexone | 44 | **-0.04 (0.03)** | 44 | **-0.17 (0.04)** |
| Olanzapine | 2 | 0.00 (0.16) | 2 | 0.07 (0.17) |
| Ondansetron | 3 | 0.00 (0.13) | 3 | 0.00 (0.13) |
| Quetiapine | 5 | -0.08 (0.12) | 5 | 0.07 (0.10) |
| Rimonabant | 1 | -0.14 (0.14) | 1 | **-0.23 (0.14)** |
| Ritanserin | 3 | -0.05 (0.07) | 3 | 0.00 (0.07) |
| Sertraline | 1 | -0.13 (0.28) | 1 | 0.00 (0.22) |
| Topiramate | 10 | -0.03 (0.06) | 10 | -0.04 (0.06) |
| Valproate | 3 | -0.01 (0.20) | 3 | **-0.38 (0.21)** |
| Varenicline | 4 | 0.00 (0.13) | 5 | -0.09 (0.11) |
| Zonisamide | 2 | 0.00 (0.22) | 2 | 0.00 (0.22) |

*Note*. Negative effect sizes indicate a more favorable medication effect. Bold indicates significant medication effect sizes.

**Supplemental Table 5.** Estimated effect sizes and standard errors for medications tested on percent days abstinent and percent days heavy drinking in randomized clinical trials

| **Medication** | **Number of Percent Days Abstinent Effect Sizes** | **Cohen’s *d* for Percent Days Abstinent** | **SE** | **Number of Percent Days Heavy Drinking Effect Sizes** | **Cohen’s *d* for Percent Days Heavy Drinking** | **SE** |
| --- | --- | --- | --- | --- | --- | --- |
| Acamprosate | 28 | **-0.27** | **0.08** | 28 | -0.03 | 0.06 |
| Aripiprazole | 1 | 0.13 | 0.12 | 1 | 0.00 | 0.13 |
| Baclofen | 12 | -0.12 | 0.13 | 12 | -0.03 | 0.13 |
| Carbamazepine | 1 | 0.00 | 0.38 | 1 | 0.00 | 0.38 |
| Gabapentin | 8 | -0.01 | 0.11 | 8 | **-0.63** | **0.29** |
| Levetiracetam | 3 | 0.01 | 0.10 | 3 | -0.15 | 0.21 |
| Memantine | 1 | 0.78 | 0.35 | 1 | 0.79 | 0.35 |
| Nalmefene | 7 | 0.00 | 0.05 | 9 | **-0.30** | **0.04** |
| Naltrexone | 45 | **-0.35** | **0.08** | 48 | **-0.09** | **0.04** |
| Olanzapine | 2 | -0.11 | 0.45 | 2 | 0.00 | 0.16 |
| Ondansetron | 3 | **-0.19** | **0.13** | 4 | -0.03 | 0.11 |
| Quetiapine | 5 | **-0.18** | **0.17** | 5 | **-0.21** | **0.17** |
| Rimonabant | 1 | -0.09 | 0.13 | 1 | 0.00 | 0.13 |
| Ritanserin | 3 | 0.03 | 0.29 | 3 | 0.00 | 0.07 |
| Sertraline | 1 | 0.00 | 0.22 | 1 | 0.00 | 0.22 |
| Topiramate | 10 | **-0.34** | **0.11** | 12 | **-0.44** | **0.11** |
| Valproate | 3 | 0.00 | 0.19 | 3 | **-0.28** | **0.19** |
| Varenicline | 4 | **-0.15** | **0.10** | 4 | **-0.19** | **0.13** |
| Zonisamide | 2 | **-0.45** | **0.43** | 2 | -0.49 | 0.50 |

*Note*. Negative effect sizes indicate a more favorable medication effect. Bold indicates significant medication effect sizes.

**Supplemental Table 6.** Estimated effect sizes and standard errors for medications tested on drinks per day and drinks per drinking day in randomized clinical trials

| **Medication** | **Number of Drinks per Day Effect Sizes** | **Cohen’s *d* for Drinks per Day** | **SE** | **Number of Drinks per Drinking Day Effect Sizes** | **Cohen’s *d* for Drinks per Drinking Day** | **SE** |
| --- | --- | --- | --- | --- | --- | --- |
| Acamprosate | 28 | 0.00 | 0.06 | 28 | 0.00 | 0.06 |
| Aripiprazole | 1 | 0.00 | 0.13 | 1 | **-0.37** | **0.12** |
| Baclofen | 9 | -0.03 | 0.10 | 9 | 0.32 | 0.22 |
| Carbamazepine |  |  |  | 1 | 0.00 | 0.38 |
| Gabapentin | 8 | **-0.23** | **0.15** | 8 | -0.08 | 0.10 |
| Levetiracetam | 3 | 0.00 | 0.29 | 3 | 0.06 | 0.10 |
| Memantine | 1 | 0.00 | 0.34 | 1 | 0.00 | 0.34 |
| Nalmefene | 9 | **-0.23** | **0.04** | 7 | -0.04 | 0.05 |
| Naltrexone | 47 | **-0.07** | **0.06** | 46 | **-0.06** | **0.03** |
| Olanzapine | 2 | 0.09 | 0.16 | 2 | 0.04 | 0.16 |
| Ondansetron | 4 | **-0.24** | **0.12** | 4 | **-0.30** | **0.12** |
| Quetiapine | 5 | -0.01 | 0.09 | 5 | -0.01 | 0.09 |
| Rimonabant | 1 | 0.00 | 0.13 | 1 | -0.07 | 0.13 |
| Ritanserin | 3 | -0.26 | 0.35 | 3 | -0.10 | 0.20 |
| Sertraline | 1 | 0.00 | 0.22 | 1 | 0.00 | 0.22 |
| Topiramate | 11 | **-0.31** | **0.24** | 11 | **-0.22** | **0.07** |
| Valproate |  |  |  | 3 | **-0.35** | **0.19** |
| Varenicline | 4 | **-0.14** | **0.10** | 4 | **-0.19** | **0.10** |
| Zonisamide | 2 | -0.41 | 0.41 | 2 | 0.00 | 0.22 |

*Note*. Negative effect sizes indicate a more favorable medication effect. Bold indicates significant medication effect sizes.

**Supplemental Figure Captions**

**Supplemental Figure 1.** Williamson-York regression models with preclinical (2-BC alcohol consumption [Panel A], 2-BC alcohol preference [Panel B], and reinstatement [Panel C]; x-axis) medication effect sizes predicting medication effect sizes on cue-induced alcohol craving in the human laboratory (y-axis). Each medication appears as a dot on the regression line, with smaller dots indicating more error variance and larger dots indicating less error variance around each estimate. Negative effect sizes represent a favorable medication effect relative to placebo/active control.

**Supplemental Figure 2.** Williamson-York regression models with preclinical (2-BC alcohol consumption [Panel A], 2-BC alcohol preference [Panel B], and reinstatement [Panel C]; x-axis) medication effect sizes predicting medication effect sizes on the percent days abstinent RCT endpoint (y-axis). Each medication appears as a dot on the regression line, with smaller dots indicating more error variance and larger dots indicating less error variance around each estimate. Negative effect sizes represent a favorable medication effect relative to placebo/active control.

**Supplemental Figure 3.** Williamson-York regression models with preclinical (2-BC alcohol consumption [Panel A], 2-BC alcohol preference [Panel B], and reinstatement [Panel C]; x-axis) medication effect sizes predicting medication effect on percent heavy drinking days RCT endpoint; y-axis). Each medication appears as a dot on the regression line, with smaller dots indicating more error variance and larger dots indicating less error variance around each estimate. Negative effect sizes represent a favorable medication effect relative to placebo/active control.

**Supplemental Figure 4.** Williamson-York regression models with preclinical (2-BC alcohol consumption [Panel A], 2-BC alcohol preference [Panel B], and reinstatement [Panel C]; x-axis) medication effect sizes predicting medication effect on drinks per day RCT endpoint; y-axis). Each medication appears as a dot on the regression line, with smaller dots indicating more error variance and larger dots indicating less error variance around each estimate. Negative effect sizes represent a favorable medication effect relative to placebo/active control.

**Supplemental Figure 5.** Williamson-York regression models with preclinical (2-BC alcohol consumption [Panel A], 2-BC alcohol preference [Panel B], and reinstatement [Panel C]; x-axis) medication effect sizes predicting medication effect on the drinks per drinking day RCT endpoint; y-axis). Each medication appears as a dot on the regression line, with smaller dots indicating more error variance and larger dots indicating less error variance around each estimate. Negative effect sizes represent a favorable medication effect relative to placebo/active control.

**Supplemental Figure 1A**

**
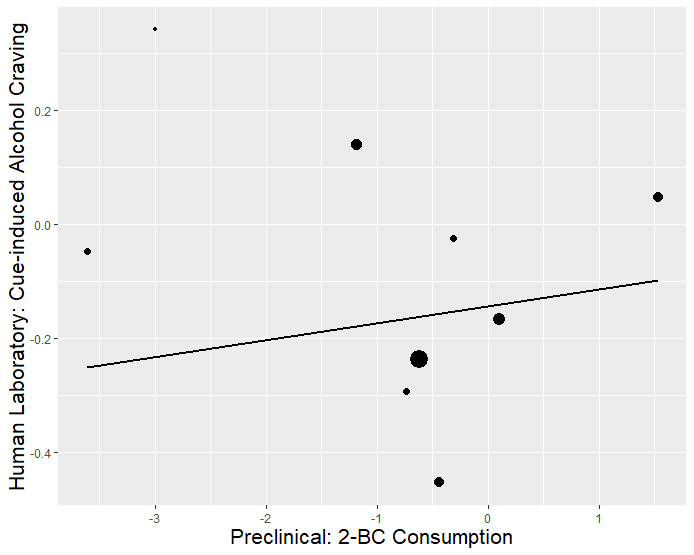
**

**Supplemental Figure 1B**

**
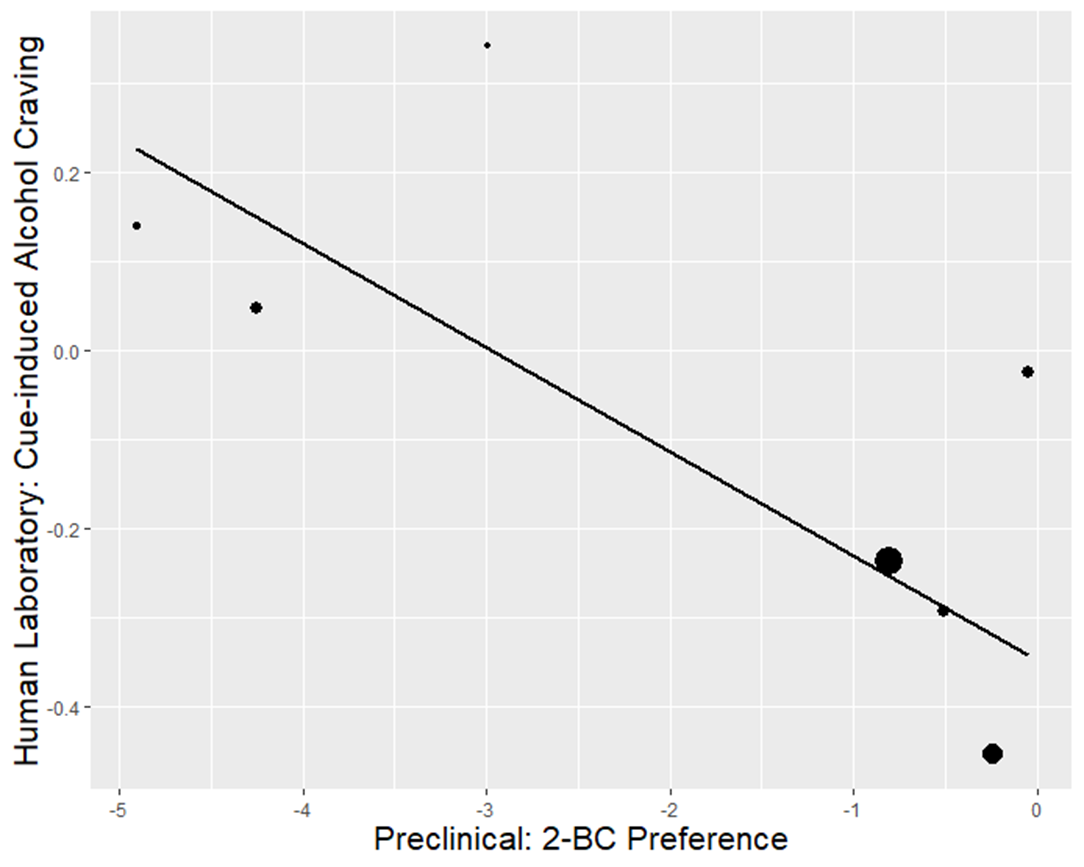
**

**Supplemental Figure 1C**

**
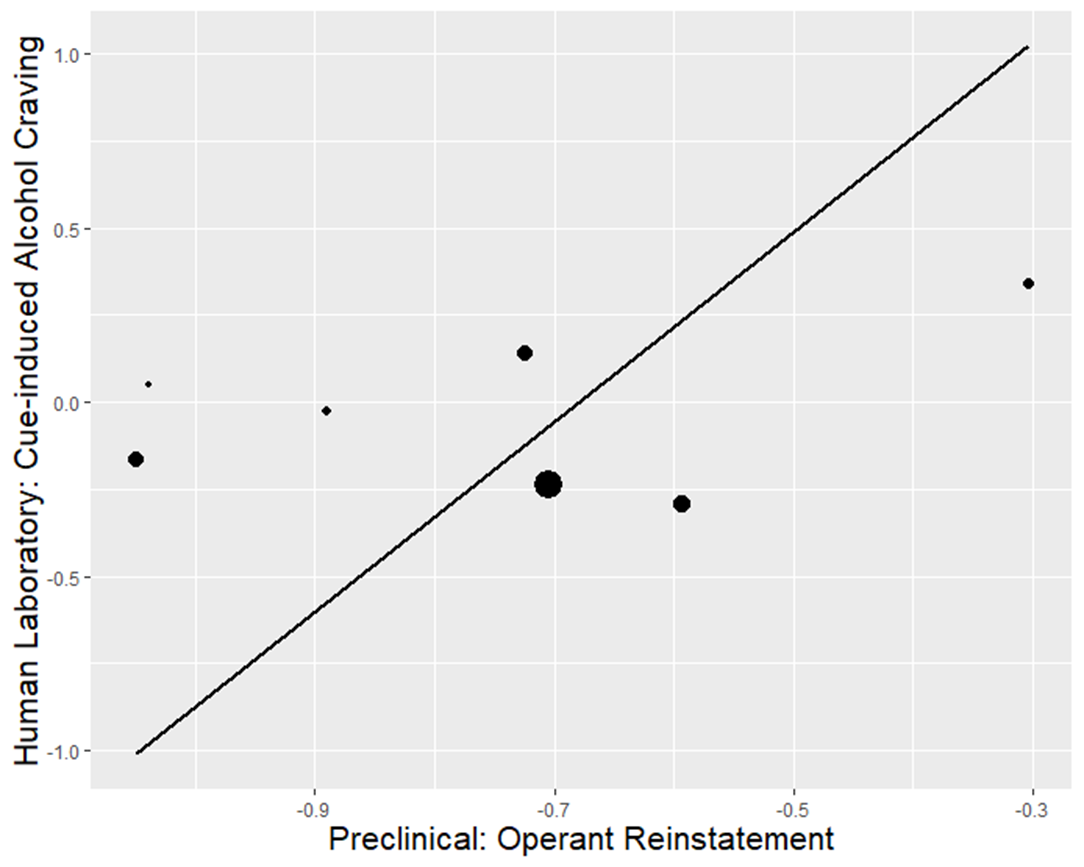
**

**Supplemental Figure 2A**

**
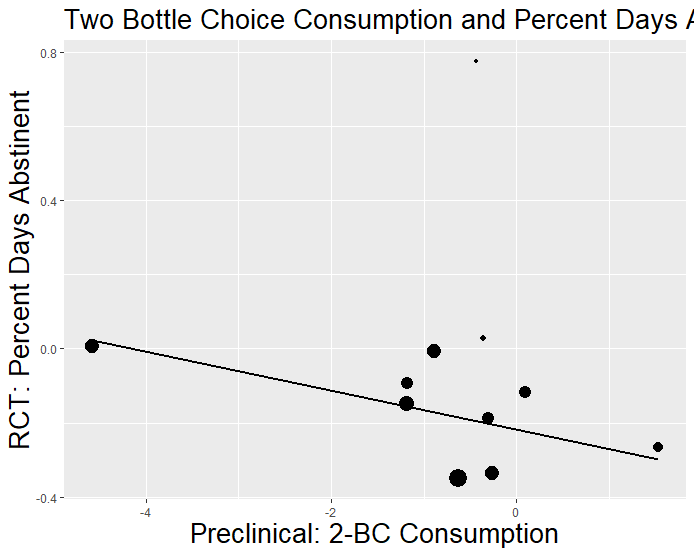
**

**Supplemental Figure 2B**

**
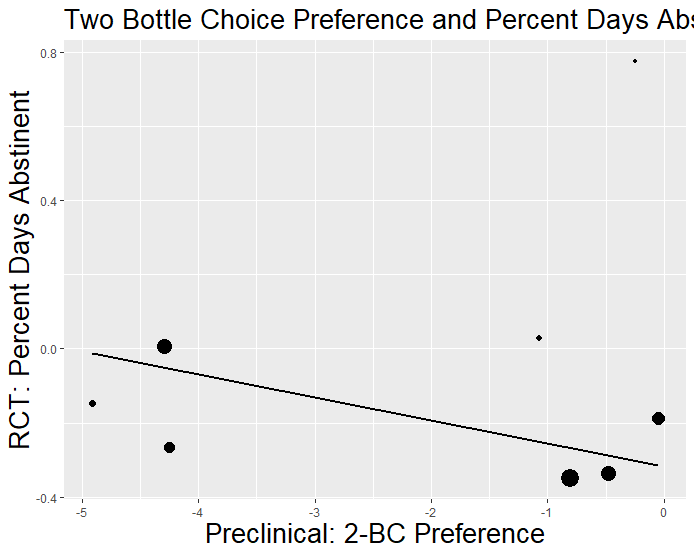
**

**Supplemental Figure 2C**

**
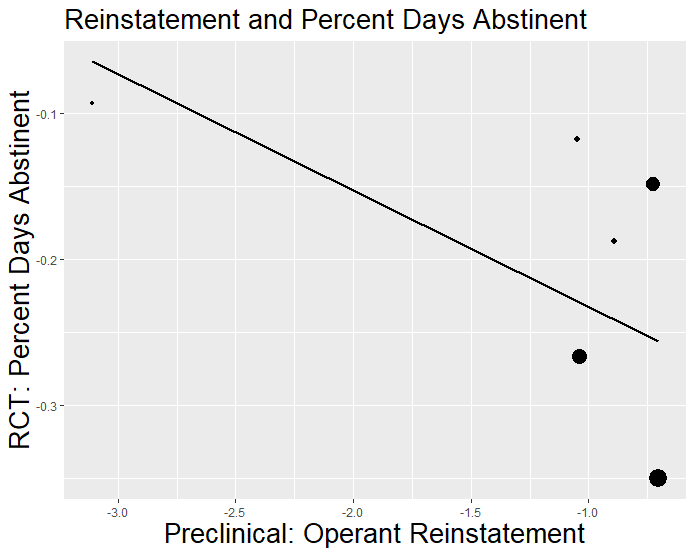
**

**Supplemental Figure 3A**

**
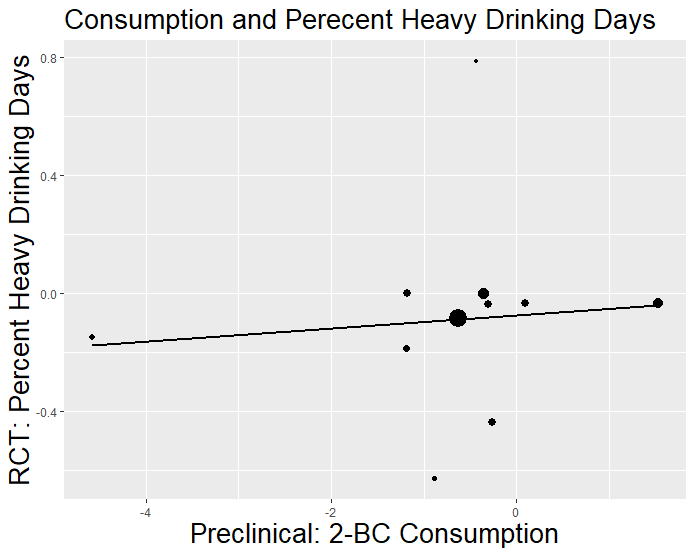
**

**Supplemental Figure 3B**

**
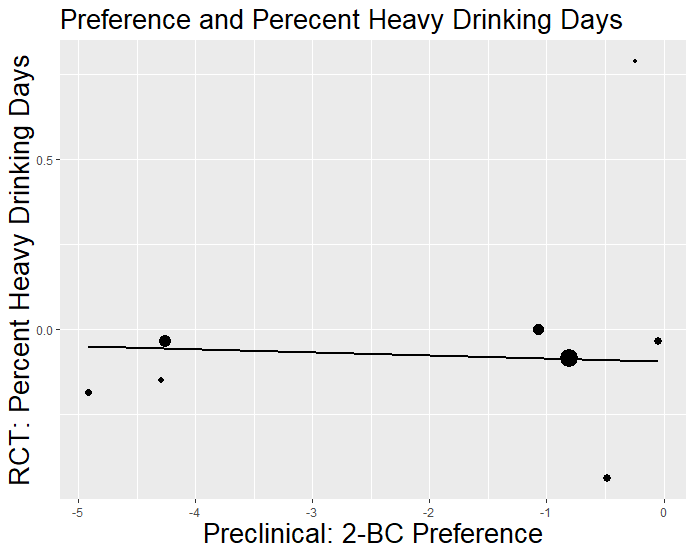
**

**Supplemental Figure 3C**

**
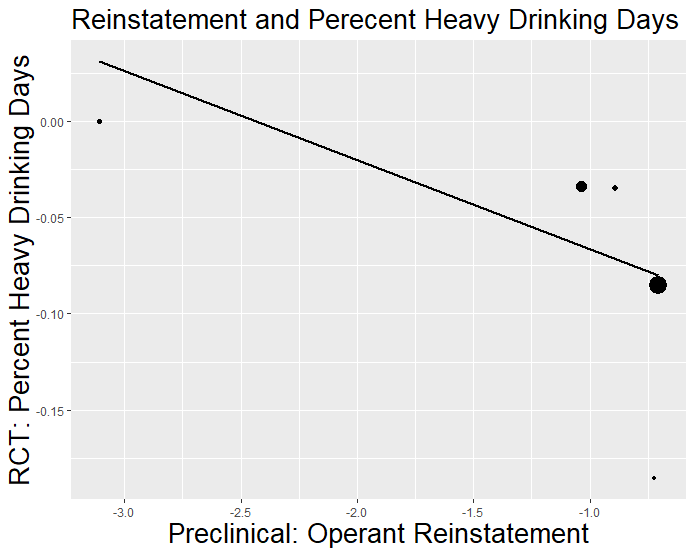
**

**Supplemental Figure 4A**

**
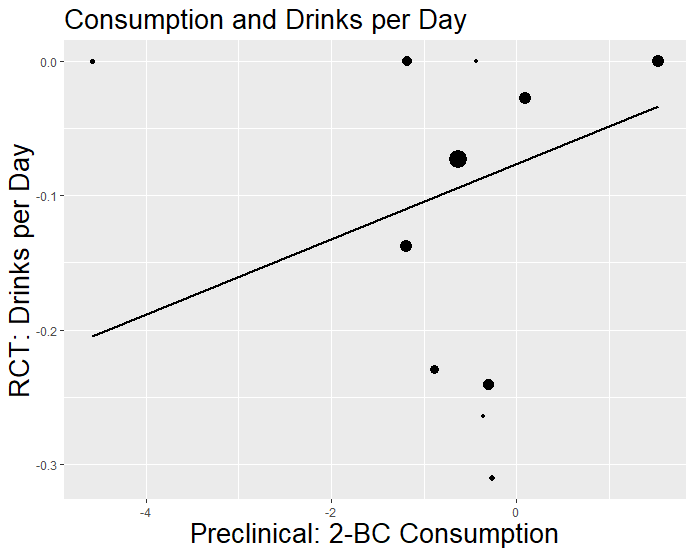
**

**Supplemental Figure 4B**

**
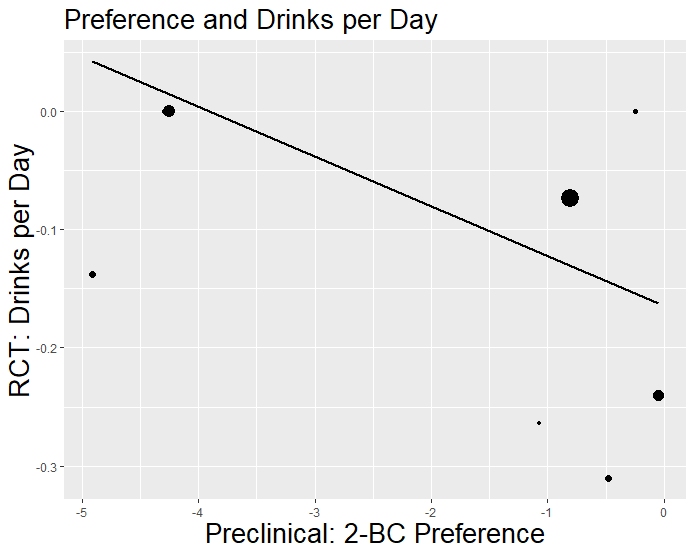
**

**Supplemental Figure 4C**

**
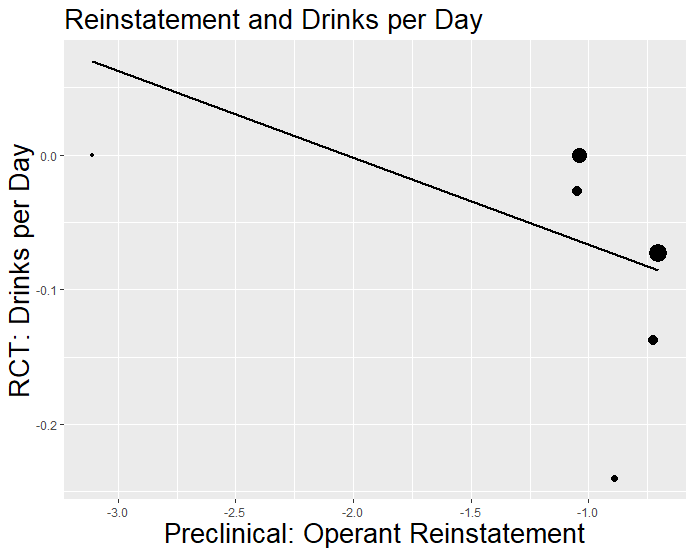
**

**Supplemental Figure 5A**

**
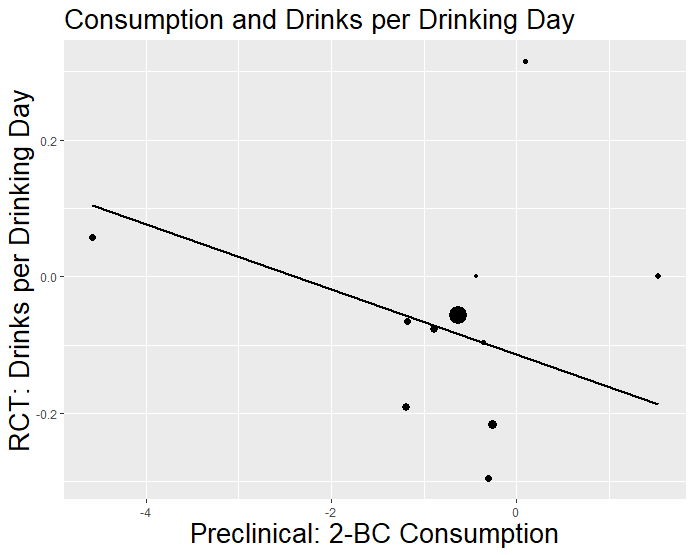
**

**Supplemental Figure 5B**

**
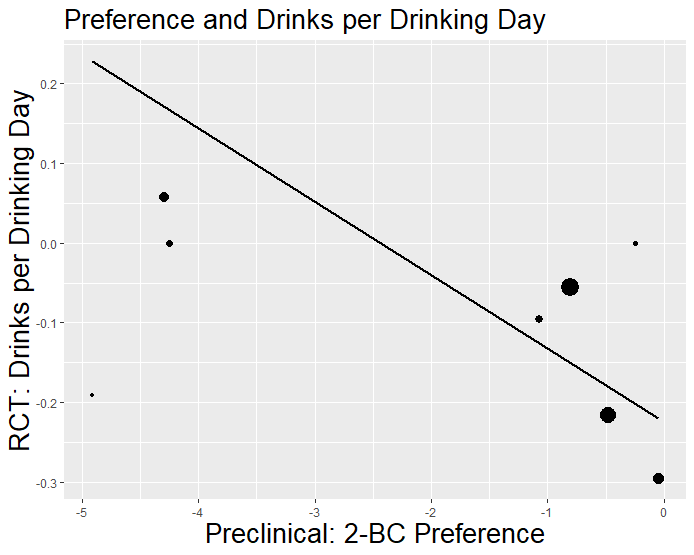
**

**Supplemental Figure 5C**

**
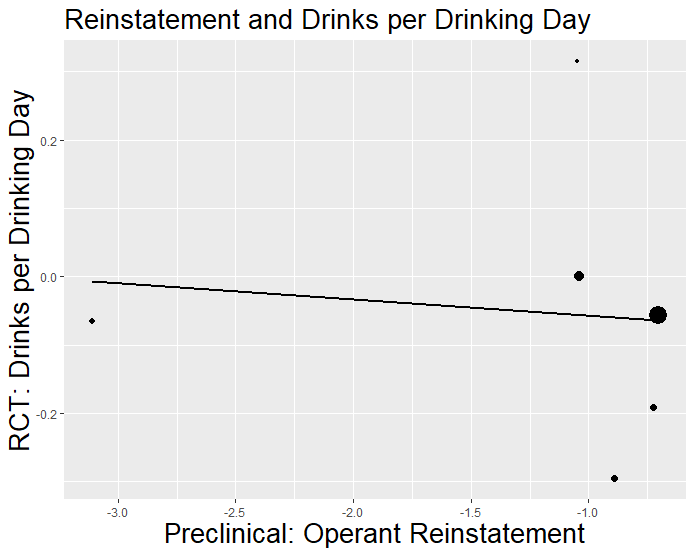
**

**Included preclinical studies**

Bachteler D, Economidou D, Danysz W, Ciccocioppo R, Spanagel R. The effects of acamprosate and neramexane on cue-induced reinstatement of ethanol-seeking behavior in rat. Neuropsychopharmacology. 2005;30(6):1104–10.

Bell RL, Lopez MF, Cui C, Egli M, Johnson KW, Franklin KM, et al. Ibudilast reduces alcohol drinking in multiple animal models of alcohol dependence. Addict Biol. 2015;20(1):38–42.

Berquist MD, Fantegrossi WE. Effects of 5-HT2A receptor agonist 2,5-dimethoxy-4-iodoamphetamine on alcohol consumption in Long-Evans rats. Behav Pharmacol. 2021;32(5):382–91.

Blednov YA, Harris RA. Metabotropic glutamate receptor 5 (mGluR5) regulation of ethanol sedation, dependence and consumption: relationship to acamprosate actions. Int J Neuropsychopharmacol. 2008;11(6):775–93.

Brager AJ, Prosser RA, Glass JD. Circadian and acamprosate modulation of elevated ethanol drinking in mPer2 clock gene mutant mice. Chronobiol Int. 2011;28(8):664–72.

Breslin FJ, Johnson BA, Lynch WJ. Effect of topiramate treatment on ethanol consumption in rats. Psychopharmacology (Berl). 2010;207(4):529–34.

Burattini C, Gill TM, Aicardi G, Janak PH. The ethanol self-administration context as a reinstatement cue: acute effects of naltrexone. Neuroscience. 2006;139(3):877–87.

Ciccocioppo R, Lin D, Martin-Fardon R, Weiss F. Reinstatement of ethanol-seeking behavior by drug cues following single versus multiple ethanol intoxication in the rat: effects of naltrexone. Psychopharmacology (Berl). 2003;168(1–2):208–15.

Ciccocioppo R, Stopponi S, Economidou D, Kuriyama M, Kinoshita H, Heilig M, et al. Chronic treatment with novel brain-penetrating selective NOP receptor agonist MT-7716 reduces alcohol drinking and seeking in the rat. Neuropsychopharmacology. 2014;39(11):2601–10.

Colombo G, Serra S, Brunetti G, Atzori G, Pani M, Vacca G, et al. The GABA(B) receptor agonists baclofen and CGP 44532 prevent acquisition of alcohol drinking behaviour in alcohol-preferring rats. Alcohol Alcohol. 2002;37(5):499–503.

Colombo G, Serra S, Vacca G, Carai MA, Gessa GL. Effect of the combination of naltrexone and baclofen, on acquisition of alcohol drinking behavior in alcohol-preferring rats. Drug Alcohol Depend. 2005;77(1):87–91.

Colombo G, Serra S, Vacca G, Gessa GL, Carai MA. Suppression by baclofen of the stimulation of alcohol intake induced by morphine and WIN 55,212-2 in alcohol-preferring rats. Eur J Pharmacol. 2004;492(2–3):189–93.

Cowen MS, Adams C, Kraehenbuehl T, Vengeliene V, Lawrence AJ. The acute anti-craving effect of acamprosate in alcohol-preferring rats is associated with modulation of the mesolimbic dopamine system. Addict Biol. 2005;10(3):233–42.

Cramer CM, Gardell LR, Boedeker KL, Harris JR, Hubbell CL, Reid LD. Isradipine combined with naltrexone persistently reduces the reward-relevant effects of cocaine and alcohol. Pharmacol Biochem Behav. 1998;60(2):345–56.

Critcher EC, Lin CI, Patel J, Myers RD. Attenuation of alcohol drinking in tetrahydroisoquinoline-treated rats by morphine and naltrexone. Pharmacol Biochem Behav. 1983;18(2):225–9.

Dayas CV, Liu X, Simms JA, Weiss F. Distinct patterns of neural activation associated with ethanol seeking: effects of naltrexone. Biol Psychiatry. 2007;61(8):979–89.

Dyr W, Ligieza J, Kostowski W. The effect of cannabinoid CB(1) receptor antagonist rimonabant (SR-141716) on ethanol drinking in high-preferring rats. Alcohol. 2008;42(6):509–12.

Farook JM, Lewis B, Littleton JM, Barron S. Topiramate attenuates the stress-induced increase in alcohol consumption and preference in male C57BL/6J mice. Physiol Behav. 2009;96(1):189–93.

Froehlich JC, Fischer SM, Dilley JE, Nicholson ER, Smith TN, Filosa NJ, et al. Combining Varenicline (Chantix) with Naltrexone Decreases Alcohol Drinking More Effectively Than Does Either Drug Alone in a Rodent Model of Alcoholism. Alcohol Clin Exp Res. 2016;40(9):1961–70.

Froehlich JC, Fischer SM, Nicholson ER, Dilley JE, Filosa NJ, Smith TN, et al. A Combination of NaltrexoneÂ +Â Varenicline Retards the Expression of a Genetic Predisposition Toward High Alcohol Drinking. Alcohol Clin Exp Res. 2017;41(3):644–52.

Froehlich JC, Hausauer BJ, Federoff DL, Fischer SM, Rasmussen DD. Prazosin reduces alcohol drinking throughout prolonged treatment and blocks the initiation of drinking in rats selectively bred for high alcohol intake. Alcohol Clin Exp Res. 2013;37(9):1552–60.

Froehlich JC, Hausauer BJ, Rasmussen DD. Combining naltrexone and prazosin in a single oral medication decreases alcohol drinking more effectively than does either drug alone. Alcohol Clin Exp Res. 2013;37(10):1763–70.

Funk D, Coen K, Tamadon S, LÃª AD. Effects of the Alpha-1 Antagonist Prazosin on KOR Agonist-Induced Reinstatement of Alcohol Seeking. Int J Neuropsychopharmacol. 2019;22(11):724–34.

Gabriel KI, Cunningham CL. Effects of topiramate on ethanol and saccharin consumption and preferences in C57BL/6J mice. Alcohol Clin Exp Res. 2005;29(1):75–80.

Hargreaves GA, McGregor IS. Topiramate moderately reduces the motivation to consume alcohol and has a marked antidepressant effect in rats. Alcohol Clin Exp Res. 2007;31(11):1900–7.

Hay RA, Jennings JH, Zitzman DL, Hodge CW, Robinson DL. Specific and nonspecific effects of naltrexone on goal-directed and habitual models of alcohol seeking and drinking. Alcohol Clin Exp Res. 2013;37(7):1100–10.

JuÃ¡rez J, Barrios De Tomasi E. Naltrexone treatment produces dose-related effects on food and water intake but daily alcohol consumption is not affected. Nutr Neurosci. 2008;11(4):183–92.

Kamens HM, Andersen J, Picciotto MR. Modulation of ethanol consumption by genetic and pharmacological manipulation of nicotinic acetylcholine receptors in mice. Psychopharmacology (Berl). 2010;208(4):613–26.

Kemppainen H, Raivio N, Kiianmaa K. Role for ventral pallidal GABAergic mechanisms in the regulation of ethanol self-administration. Psychopharmacology (Berl). 2012;223(2):211–21.

Kornet M, Goosen C, Van Ree JM. Effect of naltrexone on alcohol consumption during chronic alcohol drinking and after a period of imposed abstinence in free-choice drinking rhesus monkeys. Psychopharmacology (Berl). 1991;104(3):367–76.

Kosten TA. Pharmacologically targeting the P2rx4 gene on maintenance and reinstatement of alcohol self-administration in rats. Pharmacol Biochem Behav. 2011;98(4):533–8.

Li J, Cheng Y, Bian W, Liu X, Zhang C, Ye JH. Region-specific induction of FosB/Î”FosB by voluntary alcohol intake: effects of naltrexone. Alcohol Clin Exp Res. 2010;34(10):1742–50.

Li J, Xu K, Ding H, Xi Q. Gabapentin Reduces Alcohol Intake in Rats by Regulating NF-ÎºB Signaling Pathway Via PPAR Î³. Alcohol Alcohol. 2022;57(2):234–41.

LidÃ¶ HH, Marston H, Ericson M, SÃ¶derpalm B. The glycine reuptake inhibitor Org24598 and acamprosate reduce ethanol intake in the rat; tolerance development to acamprosate but not to Org24598. Addict Biol. 2012;17(5):897–907.

Lin N, Hubbard JI. The increased ethanol preference in rats induced by choice, darkness, or drugs is reduced by ritanserin. Brain Res Bull. 1994;33(6):633–8.

Lynch WJ, Bond C, Breslin FJ, Johnson BA. Severity of drinking as a predictor of efficacy of the combination of ondansetron and topiramate in rat models of ethanol consumption and relapse. Psychopharmacology (Berl). 2011;217(1):3–12.

LÃª AD, Funk D, Harding S, Juzytsch W, Fletcher PJ, Shaham Y. Effects of dexfenfluramine and 5-HT3 receptor antagonists on stress-induced reinstatement of alcohol seeking in rats. Psychopharmacology (Berl). 2006;186(1):82–92.

LÃª AD, Funk D, Juzytsch W, Coen K, Navarre BM, Cifani C, et al. Effect of prazosin and guanfacine on stress-induced reinstatement of alcohol and food seeking in rats. Psychopharmacology (Berl). 2011;218(1):89–99.

LÃª AD, Poulos CX, Harding S, Watchus J, Juzytsch W, Shaham Y. Effects of naltrexone and fluoxetine on alcohol self-administration and reinstatement of alcohol seeking induced by priming injections of alcohol and exposure to stress. Neuropsychopharmacology. 1999;21(3):435–44.

LÃ³pez-Moreno JA, GonzÃ¡lez-Cuevas G, Navarro M. The CB1 cannabinoid receptor antagonist rimonabant chronically prevents the nicotine-induced relapse to alcohol. Neurobiol Dis. 2007;25(2):274–83.

Maccioni P, Bienkowski P, Carai MA, Gessa GL, Colombo G. Baclofen attenuates cue-induced reinstatement of alcohol-seeking behavior in Sardinian alcohol-preferring (sP) rats. Drug Alcohol Depend. 2008;95(3):284–7.

Malpass GE, Williams HL, McMillen BA. Effects of the non-competitive NMDA receptor antagonist memantine on the volitional consumption of ethanol by alcohol-preferring rats. Basic Clin Pharmacol Toxicol. 2010;106(5):435–44.

Meert TF. Effects of various serotonergic agents on alcohol intake and alcohol preference in Wistar rats selected at two different levels of alcohol preference. Alcohol Alcohol. 1993;28(2):157–70.

Middaugh LD, Bandy AL. Naltrexone effects on ethanol consumption and response to ethanol conditioned cues in C57BL/6 mice. Psychopharmacology (Berl). 2000;151(4):321–7.

Minnaard AM, Ramakers GMJ, Vanderschuren L, Lesscher HMB. Baclofen and naltrexone, but not N-acetylcysteine, affect voluntary alcohol drinking in rats regardless of individual levels of alcohol intake. Behav Pharmacol. 2021;32(2&3):251–7.

Myers RD, Lankford MF. Failure of the 5-HT2 receptor antagonist, ritanserin, to alter preference for alcohol in drinking rats. Pharmacol Biochem Behav. 1993;45(1):233–7.

Myers RD, Lankford MF. Suppression of alcohol preference in high alcohol drinking rats: efficacy of amperozide versus naltrexone. Neuropsychopharmacology. 1996;14(2):139–49.

MÃ©ndez-DÃ­az M, Caynas Rojas S, GÃ³mez Armas D, Ruiz-Contreras AE, Aguilar-Roblero R, ProspÃ©ro-GarcÃ­a O. Endocannabinoid/GABA interactions in the entopeduncular nucleus modulates alcohol intake in rats. Brain Res Bull. 2013;91:31–7.

Naassila M, BeaugÃ© FJ, SÃ©bire N, Daoust M. Intracerebroventricular injection of antisense oligos to nNOS decreases rat ethanol intake. Pharmacol Biochem Behav. 2000;67(3):629–36.

Naassila M, Legrand E, d’Alche-BirÃ©e F, Daoust M. Cyamemazine decreases ethanol intake in rats and convulsions during ethanol withdrawal syndrome in mice. Psychopharmacology (Berl). 1998;140(4):421–8.

Nguyen SA, Malcolm R, Middaugh LD. Topiramate reduces ethanol consumption by C57BL/6 mice. Synapse. 2007;61(3):150–6.

Nicholson ER, Dilley JE, Froehlich JC. Co-Administration of Low-Dose Naltrexone and Bupropion Reduces Alcohol Drinking in Alcohol-Preferring (P) Rats. Alcohol Clin Exp Res. 2018;42(3):571–7.

Nielsen CK, Simms JA, Pierson HB, Li R, Saini SK, Ananthan S, et al. A novel delta opioid receptor antagonist, SoRI-9409, produces a selective and long-lasting decrease in ethanol consumption in heavy-drinking rats. Biol Psychiatry. 2008;64(11):974–81.

Oberlin BG, Bristow RE, Heighton ME, Grahame NJ. Pharmacologic dissociation between impulsivity and alcohol drinking in high alcohol preferring mice. Alcohol Clin Exp Res. 2010;34(8):1363–75.

Oka M, Hirouchi M, Tamura M, Sugahara S, Oyama T. Acamprosate {monocalcium bis(3-acetamidopropane-1-sulfonate)} reduces ethanol-drinking behavior in rats and glutamate-induced toxicity in ethanol-exposed primary rat cortical neuronal cultures. Eur J Pharmacol. 2013;718(1–3):323–31.

Olive MF, Nannini MA, Ou CJ, Koenig HN, Hodge CW. Effects of acute acamprosate and homotaurine on ethanol intake and ethanol-stimulated mesolimbic dopamine release. Eur J Pharmacol. 2002;437(1–2):55–61.

Panocka I, Ciccocioppo R, Polidori C, Massi M. The nucleus accumbens is a site of action for the inhibitory effect of ritanserin on ethanol intake in rats. Pharmacol Biochem Behav. 1993;46(4):857–62.

Panocka I, Massi M. Long-lasting suppression of alcohol preference in rats following serotonin receptor blockade by ritanserin. Brain Res Bull. 1992;28(3):493–6.

Patkar OL, Belmer A, Tarren JR, Holgate JY, Bartlett SE. The effect of varenicline on binge-like ethanol consumption in mice is Î²4 nicotinic acetylcholine receptor-independent. Neurosci Lett. 2016;633:235–9.

Perfumi M, Santoni M, Cippitelli A, Ciccocioppo R, Froldi R, Massi M. Hypericum perforatum CO2 extract and opioid receptor antagonists act synergistically to reduce ethanol intake in alcohol-preferring rats. Alcohol Clin Exp Res. 2003;27(10):1554–62.

Pickering C, Liljequist S. Cue-induced behavioural activation: a novel model of alcohol craving? Psychopharmacology (Berl). 2003;168(3):307–13.

Rasmussen DD, Alexander LL, Raskind MA, Froehlich JC. The alpha1-adrenergic receptor antagonist, prazosin, reduces alcohol drinking in alcohol-preferring (P) rats. Alcohol Clin Exp Res. 2009;33(2):264–72.

Rasmussen DD, Kincaid CL, Froehlich JC. Prazosin + Naltrexone Decreases Alcohol Drinking More Effectively Than Does Either Drug Alone in P Rats with a Protracted History of Extensive Voluntary Alcohol Drinking, Dependence, and Multiple Withdrawals. Alcohol Clin Exp Res. 2015;39(9):1832–41.

Rezvani AH, Overstreet DH, Levin ED, Rosenthal DI, Kordik CP, Reitz AB, et al. Effects of atypical anxiolytic N-phenyl-2-[1-[3-(2-pyridinylethynyl)benzoyl]-4-piperidine]acetamide (JNJ-5234801) on alcohol intake in alcohol-preferring P rats. Alcohol Clin Exp Res. 2007;31(1):57–63.

Rezvani AH, Overstreet DH, Mason GA, Janowsky DS, Hamedi M, Clark E, et al. Combination pharmacotherapy: a mixture of small doses of naltrexone, fluoxetine, and a thyrotropin-releasing hormone analogue reduces alcohol intake in three strains of alcohol-preferring rats. Alcohol Alcohol. 2000;35(1):76–83.

Sabino V, Kwak J, Rice KC, Cottone P. Pharmacological characterization of the 20% alcohol intermittent access model in Sardinian alcohol-preferring rats: a model of binge-like drinking. Alcohol Clin Exp Res. 2013;37(4):635–43.

Silva J, Carry E, Xue C, Zhang J, Liang J, Roberge JY, et al. A Novel Dual Drug Approach That Combines Ivermectin and Dihydromyricetin (DHM) to Reduce Alcohol Drinking and Preference in Mice. Molecules. 2021;26(6).

Silvestre JS, Palacios JM, Fernandez AG, O’Neill MF. Comparison of effects of a range of 5-HT receptor modulators on consumption and preference for a sweetened ethanol solution in rats. J Psychopharmacol. 1998;12(2):168–76.

Skelly MJ, Weiner JL. Chronic treatment with prazosin or duloxetine lessens concurrent anxiety-like behavior and alcohol intake: evidence of disrupted noradrenergic signaling in anxiety-related alcohol use. Brain Behav. 2014;4(4):468–83.

Smith BR, Robidoux J, Amit Z. GABAergic involvement in the acquisition of voluntary ethanol intake in laboratory rats. Alcohol Alcohol. 1992;27(3):227–31.

Sotomayor-ZÃ¡rate R, Gysling K, Busto UE, Cassels BK, Tampier L, Quintanilla ME. Varenicline and cytisine: two nicotinic acetylcholine receptor ligands reduce ethanol intake in University of Chile bibulous rats. Psychopharmacology (Berl). 2013;227(2):287–98.

Steensland P, Simms JA, Holgate J, Richards JK, Bartlett SE. Varenicline, an alpha4beta2 nicotinic acetylcholine receptor partial agonist, selectively decreases ethanol consumption and seeking. Proc Natl Acad Sci U S A. 2007;104(30):12518–23.

Stopponi S, de Guglielmo G, Somaini L, Cippitelli A, Cannella N, Kallupi M, et al. Activation of PPARÎ³ by pioglitazone potentiates the effects of naltrexone on alcohol drinking and relapse in msP rats. Alcohol Clin Exp Res. 2013;37(8):1351–60.

Stromberg MF, Casale M, Volpicelli L, Volpicelli JR, O’Brien CP. A comparison of the effects of the opioid antagonists naltrexone, naltrindole, and beta-funaltrexamine on ethanol consumption in the rat. Alcohol. 1998;15(4):281–9.

Stromberg MF, Mackler SA, Volpicelli JR, O’Brien CP. Effect of acamprosate and naltrexone, alone or in combination, on ethanol consumption. Alcohol. 2001;23(2):109–16.

Stromberg MF. The effect of baclofen alone and in combination with naltrexone on ethanol consumption in the rat. Pharmacol Biochem Behav. 2004;78(4):743–50.

Svensson L, Fahlke C, HÃ¥rd E, Engel JA. Involvement of the serotonergic system in ethanol intake in the rat. Alcohol. 1993;10(3):219–24.

Szczepanska R, Harding S, Grupp LA. Characterization of the decline in alcohol consumption by aminopeptidase inhibition. Drug Alcohol Depend. 1996;43(3):133–41.

SÃ¶derpalm B, Danielsson K, de Bejczy A, Adermark L, Ericson M. Combined administration of varenicline and bupropion produces additive effects on accumbal dopamine and abolishes the alcohol deprivation effect in rats. Addict Biol. 2020;25(5):e12807.

Tomkins DM, Le AD, Sellers EM. Effect of the 5-HT3 antagonist ondansetron on voluntary ethanol intake in rats and mice maintained on a limited access procedure. Psychopharmacology (Berl). 1995;117(4):479–85.

Trovero F, David S, Bernard P, Puech A, Bizot JC, Tassin JP. The Combination of Marketed Antagonists of Î±1b-Adrenergic and 5-HT2A Receptors Inhibits Behavioral Sensitization and Preference to Alcohol in Mice: A Promising Approach for the Treatment of Alcohol Dependence. PLoS One. 2016;11(3):e0151242.

Vengeliene V, Takahashi TT, Dravolina OA, Belozertseva I, Zvartau E, Bespalov AY, et al. Efficacy and side effects of baclofen and the novel GABA(B) receptor positive allosteric modulator CMPPE in animal models for alcohol and cocaine addiction. Psychopharmacology (Berl). 2018;235(7):1955–65.

Walker BM, Ehlers CL. Appetitive motivational experience during adolescence results in enhanced alcohol consumption during adulthood. Behav Neurosci. 2009;123(4):926–35.

Wang J, Blasio A, Chapman HL, Doebelin C, Liaw V, Kuryatov A, et al. Promoting activity of (Î±4)(3)(Î²2)(2) nicotinic cholinergic receptors reduces ethanol consumption. Neuropsychopharmacology. 2020;45(2):301–8.

Williams KL, Schimmel JS. Effect of naltrexone during extinction of alcohol-reinforced responding and during repeated cue-conditioned reinstatement sessions in a cue exposure style treatment. Alcohol. 2008;42(7):553–63.

Wouda JA, Riga D, De Vries W, Stegeman M, van Mourik Y, Schetters D, et al. Varenicline attenuates cue-induced relapse to alcohol, but not nicotine seeking, while reducing inhibitory response control. Psychopharmacology (Berl). 2011;216(2):267–77.

Yardley MM, Huynh N, Rodgers KE, Alkana RL, Davies DL. Oral delivery of ivermectin using a fast dissolving oral film: Implications for repurposing ivermectin as a pharmacotherapy for alcohol use disorder. Alcohol. 2015;49(6):553–9.

Zalewska-Kaszubska J, Bajer B, Czarnecka E, Dyr W, Gorska D. Voluntary alcohol consumption and plasma beta-endorphin levels in alcohol preferring rats chronically treated with levetiracetam: a preliminary study. Physiol Behav. 2011;102(5):538–41.

Zalewska-Kaszubska J, Gorska D, Dyr W, Czarnecka E. Voluntary alcohol consumption and plasma beta-endorphin levels in alcohol-preferring rats chronically treated with naltrexone. Physiol Behav. 2008;93(4–5):1005–10.

Zhou Y, Crowley RS, Ben K, Prisinzano TE, Kreek MJ. Synergistic blockade of alcohol escalation drinking in mice by a combination of novel kappa opioid receptor agonist Mesyl Salvinorin B and naltrexone. Brain Res. 2017;1662:75–86.

Zhou Y, Rubinstein M, Low MJ, Kreek MJ. Hypothalamic-specific proopiomelanocortin deficiency reduces alcohol drinking in male and female mice. Genes Brain Behav. 2017;16(4):449–61.

van Rijn RM, Whistler JL. The delta(1) opioid receptor is a heterodimer that opposes the actions of the delta(2) receptor on alcohol intake. Biol Psychiatry. 2009;66(8):777–84.

**Excluded preclinical studies**

**Note: ineligible comparator = no placebo group, ineligible medication = medication was not tested in human laboratory cue-reactivity study or RCT; ineligible outcome = outcome measured was not 2-BC consumption or preference, nor lever presses during reinstatement; ineligible study design = study did not meet the predefined inclusion criteria; ineligible phenomena of interest = study was irrelevant to the research question of medication effects on 2-BC or reinstatement outcomes.**

Aalto J, Kiianmaa K. REM-sleep deprivation-induced increase in ethanol intake: role of brain monoaminergic neurons. Alcohol. 1986;3(6):377–81.

**Reason for exclusion: Ineligible comparator**

Alaux-Cantin S, Buttolo R, Houchi H, Jeanblanc J, Naassila M. Memantine reduces alcohol drinking but not relapse in alcohol-dependent rats. Addict Biol. 2015;20(5):890–901.

**Reason for exclusion: Ineligible outcomes**

Anstrom KK, Cromwell HC, Markowski T, Woodward DJ. Effect of baclofen on alcohol and sucrose self-administration in rats. Alcohol Clin Exp Res. 2003;27(6):900–8.

**Reason for exclusion: Ineligible outcomes**

Asatryan L, Yardley MM, Khoja S, Trudell JR, Hyunh N, Louie SG, et al. Avermectins differentially affect ethanol intake and receptor function: implications for developing new therapeutics for alcohol use disorders. Int J Neuropsychopharmacol. 2014;17(6):907–16.

**Reason for exclusion: Ineligible medication**

Augier E, Dulman RS, Damadzic R, Pilling A, Hamilton JP, Heilig M. The GABA(B) Positive Allosteric Modulator ADX71441 Attenuates Alcohol Self-Administration and Relapse to Alcohol Seeking in Rats. Neuropsychopharmacology. 2017;42(9):1789–99.

**Reason for exclusion: Ineligible medication**

Augier E, Flanigan M, Dulman RS, Pincus A, Schank JR, Rice KC, et al. Wistar rats acquire and maintain self-administration of 20 % ethanol without water deprivation, saccharin/sucrose fading, or extended access training. Psychopharmacology (Berl). 2014;231(23):4561–8.

**Reason for exclusion: Ineligible outcomes**

Badia-Elder NE, Mosemiller AK, Elder RL, Froehlich JC. Naloxone retards the expression of a genetic predisposition toward alcohol drinking. Psychopharmacology (Berl). 1999;144(3):205–12.

**Reason for exclusion: Ineligible medication**

Balla A, Dong B, Shilpa BM, Vemuri K, Makriyannis A, Pandey SC, et al. Cannabinoid-1 receptor neutral antagonist reduces binge-like alcohol consumption and alcohol-induced accumbal dopaminergic signaling. Neuropharmacology. 2018;131:200–8.

**Reason for exclusion: Ineligible medication**

Barson JR, Carr AJ, Soun JE, Sobhani NC, Leibowitz SF, Hoebel BG. Opioids in the nucleus accumbens stimulate ethanol intake. Physiol Behav. 2009;98(4):453–9.

**Reason for exclusion: Ineligible medication**

Berizzi AE, Perry CJ, Shackleford DM, Lindsley CW, Jones CK, Chen NA, et al. Muscarinic M(5) receptors modulate ethanol seeking in rats. Neuropsychopharmacology. 2018;43(7):1510–7.

**Reason for exclusion: Ineligible medication**

Besheer J, Frisbee S, Randall PA, Jaramillo AA, Masciello M. Gabapentin potentiates sensitivity to the interoceptive effects of alcohol and increases alcohol self-administration in rats. Neuropharmacology. 2016;101:216–24.

**Reason for exclusion: Ineligible study design**

Besheer J, Lepoutre V, Hodge CW. GABA(B) receptor agonists reduce operant ethanol self-administration and enhance ethanol sedation in C57BL/6J mice. Psychopharmacology (Berl). 2004;174(3):358–66.

**Reason for exclusion: Ineligible study design**

Bienkowski P, Krzascik P, Koros E, Kostowski W, Scinska A, Danysz W. Effects of a novel uncompetitive NMDA receptor antagonist, MRZ 2/579 on ethanol self-administration and ethanol withdrawal seizures in the rat. Eur J Pharmacol. 2001;413(1):81–9.

**Reason for exclusion: Ineligible phenomena of interest**

Bito-Onon JJ, Simms JA, Chatterjee S, Holgate J, Bartlett SE. Varenicline, a partial agonist at neuronal nicotinic acetylcholine receptors, reduces nicotine-induced increases in 20% ethanol operant self-administration in Sprague-Dawley rats. Addict Biol. 2011;16(3):440–9.

**Reason for exclusion: Ineligible study design**

Blomqvist O, Ericson M, Johnson DH, Engel JA, SÃ¶derpalm B. Voluntary ethanol intake in the rat: effects of nicotinic acetylcholine receptor blockade or subchronic nicotine treatment. Eur J Pharmacol. 1996;314(3):257–67.

**Reason for exclusion: Ineligible medication**

Bornebusch AB, Fink-Jensen A, WÃ¶rtwein G, Seeley RJ, Thomsen M. Glucagon-Like Peptide-1 Receptor Agonist Treatment Does Not Reduce Abuse-Related Effects of Opioid Drugs. eNeuro. 2019;6(2).

**Reason for exclusion: Ineligible phenomena of interest**

BÃ¤ckstrÃ¶m P, HyytiÃ¤ P. Ionotropic glutamate receptor antagonists modulate cue-induced reinstatement of ethanol-seeking behavior. Alcohol Clin Exp Res. 2004;28(4):558–65.

**Reason for exclusion: Ineligible medication**

Chatterjee S, Steensland P, Simms JA, Holgate J, Coe JW, Hurst RS, et al. Partial agonists of the Î±3Î²4* neuronal nicotinic acetylcholine receptor reduce ethanol consumption and seeking in rats. Neuropsychopharmacology. 2011;36(3):603–15.

**Reason for exclusion: Ineligible medication**

Chaudhri N, Sahuque LL, Cone JJ, Janak PH. Reinstated ethanol-seeking in rats is modulated by environmental context and requires the nucleus accumbens core. Eur J Neurosci. 2008;28(11):2288–98.

**Reason for exclusion: Ineligible study design**

Chaudhri N, Sahuque LL, Schairer WW, Janak PH. Separable roles of the nucleus accumbens core and shell in context- and cue-induced alcohol-seeking. Neuropsychopharmacology. 2010;35(3):783–91.

**Reason for exclusion: Ineligible condition**

Chen YW, Barson JR, Chen A, Hoebel BG, Leibowitz SF. Opioids in the perifornical lateral hypothalamus suppress ethanol drinking. Alcohol. 2013;47(1):31–8.

**Reason for exclusion: Ineligible medication**

Cichelli MJ, Lewis MJ. Naloxone nonselective suppression of drinking of ethanol, sucrose, saccharin, and water by rats. Pharmacol Biochem Behav. 2002;72(3):699–706.

**Reason for exclusion: Ineligible medication**

Cippitelli A, Brunori G, Schoch J, Armishaw CJ, Wu J, Zaveri NT, et al. Differential regulation of alcohol taking and seeking by antagonism at Î±4Î²2 and Î±3Î²4 nAChRs. Psychopharmacology (Berl). 2018;235(6):1745–57.

**Reason for exclusion: Ineligible medication**

Cippitelli A, Cannella N, Braconi S, Duranti A, Tontini A, Bilbao A, et al. Increase of brain endocannabinoid anandamide levels by FAAH inhibition and alcohol abuse behaviours in the rat. Psychopharmacology (Berl). 2008;198(4):449–60.

**Reason for exclusion: Ineligible study design**

Colombo G, Vacca G, Serra S, Brunetti G, Carai MA, Gessa GL. Baclofen suppresses motivation to consume alcohol in rats. Psychopharmacology (Berl). 2003;167(3):221–4.

**Reason for exclusion: Ineligible outcomes**

Cook JE, Chandler C, RÃ¼edi-Bettschen D, Taylor I, Patterson S, Platt DM. Changes in the elimination and resurgence of alcohol-maintained behavior in rats and the effects of naltrexone. Psychol Addict Behav. 2020;34(1):10–22.

**Reason for exclusion: Ineligible phenomena of interest**

Coonfield DL, Hill KG, Kaczmarek HJ, Ferraro FM, Kiefer SW. Low doses of naltrexone reduce palatability and consumption of ethanol in outbred rats. Alcohol. 2002;26(1):43–7.

**Reason for exclusion: inadequate descripte statistics**

Czachowski CL, Delory MJ. Acamprosate and naltrexone treatment effects on ethanol and sucrose seeking and intake in ethanol-dependent and nondependent rats. Psychopharmacology (Berl). 2009;204(2):335–48.

**Reason for exclusion: Ineligible study design**

Czachowski CL, Legg BH, Samson HH. Effects of acamprosate on ethanol-seeking and self-administration in the rat. Alcohol Clin Exp Res. 2001;25(3):344–50.

**Reason for exclusion: Ineligible study design**

De Witte PA, Hamon M, Mauborgne A, Cesselin F, Levy C, Laduron PM. Ethanol and opiate decrease the axonal transport of substance-P like immunoreactive material in rat vagus-nerves. Neuropeptides. 1990;16(1):15–20.

**Reason for exclusion: Ineligible medication**

Domi E, Xu L, PÃ¤tz M, Nordeman A, Augier G, Holm L, et al. Nicotine increases alcohol self-administration in male rats via a Î¼-opioid mechanism within the mesolimbic pathway. Br J Pharmacol. 2020;177(19):4516–31.

**Reason for exclusion: Ineligible medication**

Duke AN, Kaminski BJ, Weerts EM. Baclofen effects on alcohol seeking, self-administration and extinction of seeking responses in a within-session design in baboons. Addict Biol. 2014;19(1):16–26.

**Reason for exclusion: Ineligible outcomes**

Dyr W, Koros E, Bienkowski P, Kostowski W. Involvement of nicotinic acetylcholine receptors in the regulation of alcohol drinking in Wistar rats. Alcohol Alcohol. 1999;34(1):43–7.

**Reason for exclusion: Ineligible medication**

Echeverry-Alzate V, Jeanblanc J, Sauton P, Bloch V, Labat L, Soichot M, et al. Is R(+)-Baclofen the best option for the future of Baclofen in alcohol dependence pharmacotherapy? Insights from the preclinical side. Addict Biol. 2021;26(2):e12892.

**Reason for exclusion: Ineligible outcomes**

Echeverry-Alzate V, Tuda-ArÃ­zcun M, BÃ¼hler KM, Santos Ã, GinÃ© E, Olmos P, et al. Cocaine reverses the naltrexone-induced reduction in operant ethanol self-administration: the effects on immediate-early gene expression in the rat prefrontal cortex. Neuropharmacology. 2012;63(6):927–35.

**Reason for exclusion: Ineligible study design**

Eisenhardt M, Leixner S, LujÃ¡n R, Spanagel R, Bilbao A. Glutamate Receptors within the Mesolimbic Dopamine System Mediate Alcohol Relapse Behavior. J Neurosci. 2015;35(47):15523–38.

**Reason for exclusion: Ineligible medication**

Ericson M, Blomqvist O, Engel JA, SÃ¶derpalm B. Voluntary ethanol intake in the rat and the associated accumbal dopamine overflow are blocked by ventral tegmental mecamylamine. Eur J Pharmacol. 1998;358(3):189–96.

**Reason for exclusion: Ineligible medication**

Escher T, Call SB, Blaha CD, Mittleman G. Behavioral effects of aminoadamantane class NMDA receptor antagonists on schedule-induced alcohol and self-administration of water in mice. Psychopharmacology (Berl). 2006;187(4):424–34.

**Reason for exclusion: Ineligible study design**

Farook JM, Lewis B, Gaddis JG, Littleton JM, Barron S. Effects of mecamylamine on alcohol consumption and preference in male C57BL/6J mice. Pharmacology. 2009;83(6):379–84.

**Reason for exclusion: Ineligible medication**

Feduccia AA, Simms JA, Mill D, Yi HY, Bartlett SE. Varenicline decreases ethanol intake and increases dopamine release via neuronal nicotinic acetylcholine receptors in the nucleus accumbens. Br J Pharmacol. 2014;171(14):3420–31.

**Reason for exclusion: Ineligible study design**

Ford MM, Beckley EH, Nickel JD, Eddy S, Finn DA. Ethanol intake patterns in female mice: influence of allopregnanolone and the inhibition of its synthesis. Drug Alcohol Depend. 2008;97(1–2):73–85.

**Reason for exclusion: Ineligible medication**

Ford MM, Fretwell AM, Nickel JD, Mark GP, Strong MN, Yoneyama N, et al. The influence of mecamylamine on ethanol and sucrose self-administration. Neuropharmacology. 2009;57(3):250–8.

**Reason for exclusion: Ineligible medication**

Ford MM, Nickel JD, Finn DA. Treatment with and withdrawal from finasteride alter ethanol intake patterns in male C57BL/6J mice: potential role of endogenous neurosteroids? Alcohol. 2005;37(1):23–33.

**Reason for exclusion: Ineligible medication**

Foster KL, McKay PF, Seyoum R, Milbourne D, Yin W, Sarma PV, et al. GABA(A) and opioid receptors of the central nucleus of the amygdala selectively regulate ethanol-maintained behaviors. Neuropsychopharmacology. 2004;29(2):269–84.

**Reason for exclusion: Ineligible study design**

Fredriksson I, Jayaram-LindstrÃ¶m N, Wirf M, Nylander E, NystrÃ¶m E, Jardemark K, et al. Evaluation of guanfacine as a potential medication for alcohol use disorder in long-term drinking rats: behavioral and electrophysiological findings. Neuropsychopharmacology. 2015;40(5):1130–40.

**Reason for exclusion: Ineligible medication**

Froehlich JC, Harts J, Lumeng L, Li TK. Naloxone attenuates voluntary ethanol intake in rats selectively bred for high ethanol preference. Pharmacol Biochem Behav. 1990;35(2):385–90.

**Reason for exclusion: Ineligible medication**

Froehlich JC, Zweifel M, Harts J, Lumeng L, Li TK. Importance of delta opioid receptors in maintaining high alcohol drinking. Psychopharmacology (Berl). 1991;103(4):467–72.

**Reason for exclusion: Ineligible medication**

Gasparyan A, Navarrete F, Manzanares J. The administration of sertraline plus naltrexone reduces ethanol consumption and motivation in a long-lasting animal model of post-traumatic stress disorder. Neuropharmacology. 2021;189:108552.

**Reason for exclusion: Ineligible study design**

Gauvin DV, Moore KR, Holloway FA. Do rat strain differences in ethanol consumption reflect differences in ethanol sensitivity or the preparedness to learn? Alcohol. 1993;10(1):37–43.

**Reason for exclusion: Ineligible study design**

Gessa GL, Serra S, Vacca G, Carai MA, Colombo G. Suppressing effect of the cannabinoid CB1 receptor antagonist, SR147778, on alcohol intake and motivational properties of alcohol in alcohol-preferring sP rats. Alcohol Alcohol. 2005;40(1):46–53.

**Reason for exclusion: Ineligible medication**

Gilpin NW, Richardson HN, Koob GF. Effects of CRF1-receptor and opioid-receptor antagonists on dependence-induced increases in alcohol drinking by alcohol-preferring (P) rats. Alcohol Clin Exp Res. 2008;32(9):1535–42.

**Reason for exclusion: Ineligible medication**

Ginsburg BC, Lamb RJ. Effects of varenicline on ethanol- and food-maintained responding in a concurrent access procedure. Alcohol Clin Exp Res. 2013;37(7):1228–33.

**Reason for exclusion: Ineligible study design**

Giuliano C, Goodlett CR, Economidou D, GarcÃ­a-Pardo MP, Belin D, Robbins TW, et al. The Novel Î¼-Opioid Receptor Antagonist GSK1521498 Decreases Both Alcohol Seeking and Drinking: Evidence from a New Preclinical Model of Alcohol Seeking. Neuropsychopharmacology. 2015;40(13):2981–92.

**Reason for exclusion: Ineligible medication**

Grupp LA, Lau V, Harding S. The reduction in alcohol intake by the 5-HT1A agonist 8-OH DPAT and its attenuation by the alpha 2 adrenergic antagonist idazoxan correlates with blood glucose levels. Psychopharmacology (Berl). 1997;133(2):172–8.

**Reason for exclusion: Ineligible medication**

Gulley JM, McNamara C, Barbera TJ, Ritz MC, George FR. Selective serotonin reuptake inhibitors: effects of chronic treatment on ethanol-reinforced behavior in mice. Alcohol. 1995;12(3):177–81.

**Reason for exclusion: Ineligible medication**

Gupta T, Syed YM, Revis AA, Miller SA, Martinez M, Cohn KA, et al. Acute effects of acamprosate and MPEP on ethanol Drinking-in-the-Dark in male C57BL/6J mice. Alcohol Clin Exp Res. 2008;32(11):1992–8.

**Reason for exclusion: Ineligible study design**

Henderson-Redmond AN, Lowe TE, Tian XB, Morgan DJ. Increased ethanol drinking in “humanized” mice expressing the mu opioid receptor A118G polymorphism are mediated through sex-specific mechanisms. Brain Res Bull. 2018;138:12–9.

**Reason for exclusion: Ineligible study design**

Hendrickson LM, Zhao-Shea R, Tapper AR. Modulation of ethanol drinking-in-the-dark by mecamylamine and nicotinic acetylcholine receptor agonists in C57BL/6J mice. Psychopharmacology (Berl). 2009;204(4):563–72.

**Reason for exclusion: Ineligible medication**

Heyser CJ, Schulteis G, Durbin P, Koob GF. Chronic acamprosate eliminates the alcohol deprivation effect while having limited effects on baseline responding for ethanol in rats. Neuropsychopharmacology. 1998;18(2):125–33.

**Reason for exclusion: Ineligible outcomes**

Higley AE, Kiefer SW. Delta receptor antagonism, ethanol taste reactivity, and ethanol consumption in outbred male rats. Alcohol. 2006;40(3):143–50.

**Reason for exclusion: Ineligible study design**

Hill KG, Sable HJ, Ferraro Iii FM, Kiefer SW. Chronic naltrexone treatment and ethanol responsivity in outbred rats. Alcohol Clin Exp Res. 2010;34(2):272–9.

**Reason for exclusion: Ineligible study design**

Hwa LS, Kalinichev M, Haddouk H, Poli S, Miczek KA. Reduction of excessive alcohol drinking by a novel GABAB receptor positive allosteric modulator ADX71441 in mice. Psychopharmacology (Berl). 2014;231(2):333–43.

**Reason for exclusion: Ineligible medication**

HyytiÃ¤ P, Ingman K, Soini SL, Laitinen JT, Korpi ER. Effects of continuous opioid receptor blockade on alcohol intake and up-regulation of opioid receptor subtype signalling in a genetic model of high alcohol drinking. Naunyn Schmiedebergs Arch Pharmacol. 1999;360(4):391–401.

**Reason for exclusion: Ineligible medication**

HÃ¶lter SM, Danysz W, Spanagel R. Evidence for alcohol anti-craving properties of memantine. Eur J Pharmacol. 1996;314(3):R1-2.

**Reason for exclusion: Ineligible study design**

June HL, McCane SR, Zink RW, Portoghese PS, Li TK, Froehlich JC. The delta 2-opioid receptor antagonist naltriben reduces motivated responding for ethanol. Psychopharmacology (Berl). 1999;147(1):81–9.

**Reason for exclusion: Ineligible medication**

Kamens HM, Silva C, Peck C, Miller CN. Varenicline modulates ethanol and saccharin consumption in adolescent male and female C57BL/6J mice. Brain Res Bull. 2018;138:20–5.

**Reason for exclusion: Ineligible outcomes**

Kasten CR, Boehm SL. Intra-nucleus accumbens shell injections of R(+)- and S(-)-baclofen bidirectionally alter binge-like ethanol, but not saccharin, intake in C57Bl/6J mice. Behav Brain Res. 2014;272:238–47.

**Reason for exclusion: Ineligible study design**

Koistinen M, Tuomainen P, HyytiÃ¤ P, Kiianmaa K. Naltrexone suppresses ethanol intake in 6-hydroxydopamine-treated rats. Alcohol Clin Exp Res. 2001;25(11):1605–12.

**Reason for exclusion: Ineligible study design**

Lankford MF, Myers RD. Opiate and 5-HT2A receptors in alcohol drinking: preference in HAD rats is inhibited by combination treatment with naltrexone and amperozide. Alcohol. 1996;13(1):53–7.

**Reason for exclusion: Ineligible medication**

Lee MR, Hinton DJ, Wu J, Mishra PK, Port JD, Macura SI, et al. Acamprosate reduces ethanol drinking behaviors and alters the metabolite profile in mice lacking ENT1. Neurosci Lett. 2011;490(2):90–5.

**Reason for exclusion: Ineligible study design**

Li J, Bian WL, Xie GQ, Cui SZ, Wu ML, Li YH, et al. Chronic ethanol intake-induced changes in open-field behavior and calcium/calmodulin-dependent protein kinase IV expression in nucleus accumbens of rats: naloxone reversal. Acta Pharmacol Sin. 2008;29(6):646–52.

**Reason for exclusion: Ineligible medication**

Liang JH, Chen F, Krstew E, Cowen MS, Carroll FY, Crawford D, et al. The GABA(B) receptor allosteric modulator CGP7930, like baclofen, reduces operant self-administration of ethanol in alcohol-preferring rats. Neuropharmacology. 2006;50(5):632–9.

**Reason for exclusion: Ineligible study design**

LlidÃ³ A, BartolomÃ© I, Darbra S, PallarÃ¨s M. Effects of neonatal allopregnanolone manipulations and early maternal separation on adult alcohol intake and monoamine levels in ventral striatum of male rats. Horm Behav. 2016;82:11–20.

**Reason for exclusion: Ineligible exposure**

Lorrai I, Contini A, Gessa GL, Mugnaini C, Corelli F, Colombo G, et al. Operant, oral alcohol self-administration: Sex differences in Sardinian alcohol-preferring rats. Alcohol. 2019;79:147–62.

**Reason for exclusion: Ineligible medication**

Lovelock DF, Nguyen T, Van Voorhies K, Zhang Y, Besheer J. RTICBM-74 Is a Brain-Penetrant Cannabinoid Receptor Subtype 1 Allosteric Modulator that Reduces Alcohol Intake in Rats. J Pharmacol exp Ther. 2022;380(3):153–61.

**Reason for exclusion: Ineligible comparator**

LÃª AD, Funk D, Lo S, Coen K. Operant self-administration of alcohol and nicotine in a preclinical model of co-abuse. Psychopharmacology (Berl). 2014;231(20):4019–29.

**Reason for exclusion: Ineligible phenomena of interest**

Maccioni P, Lorrai I, Contini A, Leite-Morris K, Colombo G. Microinjection of baclofen and CGP7930 into the ventral tegmental area suppresses alcohol self-administration in alcohol-preferring rats. Neuropharmacology. 2018;136(Pt A):146–58.

**Reason for exclusion: Ineligible study design**

Marfaing-Jallat P, Miceli D, Le Magnen J. Decrease in ethanol consumption by naloxone in naive and dependent rats. Pharmacol Biochem Behav. 1983;18 Suppl 1:537–9.

**Reason for exclusion: Ineligible medication**

Maurel S, De Vry J, Schreiber R. Comparison of the effects of the selective serotonin-reuptake inhibitors fluoxetine, paroxetine, citalopram and fluvoxamine in alcohol-preferring cAA rats. Alcohol. 1999;17(3):195–201.

**Reason for exclusion: Ineligible medication**

Mhatre M, Pruthi R, Hensley K, Holloway F. 5-HT3 antagonist ICS 205-930 enhances naltrexone’s effects on ethanol intake. Eur J Pharmacol. 2004;491(2–3):149–56.

**Reason for exclusion: Ineligible comparator**

Milivojevic V, Covault J. Alcohol exposure during late adolescence increases drinking in adult Wistar rats, an effect that is not reduced by finasteride. Alcohol Alcohol. 2013;48(1):28–38.

**Reason for exclusion: Ineligible medication**

Miranda-Morales RS, Molina JC, Spear NE, Abate P. Participation of the endogenous opioid system in the acquisition of a prenatal ethanol-related memory: effects on neonatal and preweanling responsiveness to ethanol. Physiol Behav. 2010;101(1):153–60.

**Reason for exclusion: Ineligible medication**

Montesinos J, Gil A, Guerri C. Nalmefene Prevents Alcohol-Induced Neuroinflammation and Alcohol Drinking Preference in Adolescent Female Mice: Role of TLR4. Alcohol Clin Exp Res. 2017;41(7):1257–70.

**Reason for exclusion: Ineligible study design**

Moore EM, Boehm SL. Site-specific microinjection of baclofen into the anterior ventral tegmental area reduces binge-like ethanol intake in male C57BL/6J mice. Behav Neurosci. 2009;123(3):555–63.

**Reason for exclusion: Ineligible study design**

Myers RD, Critcher EC. Naloxone alters alcohol drinking induced in the rat by tetrahydropapaveroline (THP) infused ICV. Pharmacol Biochem Behav. 1982;16(5):827–36.

**Reason for exclusion: Ineligible medication**

Navarro M, Carvajal F, Lerma-Cabrera JM, Cubero I, Picker MJ, Thiele TE. Evidence that Melanocortin Receptor Agonist Melanotan-II Synergistically Augments the Ability of Naltrexone to Blunt Binge-Like Ethanol Intake in Male C57BL/6J Mice. Alcohol Clin Exp Res. 2015;39(8):1425–33.

**Reason for exclusion: Ineligible comparator**

OrrÃ¹ A, Fujani D, Cassina C, Conti M, Di Clemente A, Cervo L. Operant, oral alcoholic beer self-administration by C57BL/6J mice: effect of BHF177, a positive allosteric modulator of GABA(B) receptors. Psychopharmacology (Berl). 2012;222(4):685–700.

**Reason for exclusion: Ineligible phenomena of interest**

Overstreet DH, Knapp DJ, Breese GR. Drug challenges reveal differences in mediation of stress facilitation of voluntary alcohol drinking and withdrawal-induced anxiety in alcohol-preferring P rats. Alcohol Clin Exp Res. 2007;31(9):1473–81.

**Reason for exclusion: Ineligible medication**

O’Neil ML, Beckwith LE, Kincaid CL, Rasmussen DD. The Î±1-adrenergic receptor antagonist, doxazosin, reduces alcohol drinking in alcohol-preferring (P) Rats. Alcohol Clin Exp Res. 2013;37(2):202–12.

**Reason for exclusion: Ineligible medication**

Paronis CA. Ethanol self-administration in rats responding under concurrent schedules for milk or ethanol plus milk. Behav Pharmacol. 2013;24(5–6):486–95.

**Reason for exclusion: Ineligible comparator**

Perfumi M, Mattioli L, Cucculelli M, Massi M. Reduction of ethanol intake by chronic treatment with Hypericum perforatum, alone or combined with naltrexone in rats. J Psychopharmacol. 2005;19(5):448–54.

**Reason for exclusion: Ineligible study design**

Peters S, Slattery DA, Flor PJ, Neumann ID, Reber SO. Differential effects of baclofen and oxytocin on the increased ethanol consumption following chronic psychosocial stress in mice. Addict Biol. 2013;18(1):66–77.

**Reason for exclusion: Ineligible study design**

Petry NM. Benzodiazepine-GABA modulation of concurrent ethanol and sucrose reinforcement in the rat. Exp Clin Psychopharmacol. 1997;5(3):183–94.

**Reason for exclusion: Ineligible study design**

Petry NM. Ro 15-4513 selectively attenuates ethanol, but not sucrose, reinforced responding in a concurrent access procedure; comparison to other drugs. Psychopharmacology (Berl). 1995;121(2):192–203.

**Reason for exclusion: Ineligible medication**

Poznanski P, Lesniak A, Korostynski M, Szklarczyk K, Lazarczyk M, Religa P, et al. Delta-opioid receptor antagonism leads to excessive ethanol consumption in mice with enhanced activity of the endogenous opioid system. Neuropharmacology. 2017;118:90–101.

**Reason for exclusion: Ineligible medication**

Pushparaj A, Le Foll B. Involvement of the caudal granular insular cortex in alcohol self-administration in rats. Behav Brain Res. 2015;293:203–7.

**Reason for exclusion: Ineligible study design**

Quijano CardÃ© NA, Perez EE, Feinn R, Kranzler HR, De Biasi M. Antagonism of GluK1-containing kainate receptors reduces ethanol consumption by modulating ethanol reward and withdrawal. Neuropharmacology. 2021;199:108783.

**Reason for exclusion: Ineligible medication**

Quintanilla ME, Perez E, Tampier L. Baclofen reduces ethanol intake in high-alcohol-drinking University of Chile bibulous rats. Addict Biol. 2008;13(3–4):326–36.

**Reason for exclusion: Ineligible study design**

Quintanilla ME, Rivera-Meza M, Berrios-CÃ¡rcamo PA, Bustamante D, Buscaglia M, Morales P, et al. Salsolinol, free of isosalsolinol, exerts ethanol-like motivational/sensitization effects leading to increases in ethanol intake. Alcohol. 2014;48(6):551–9.

**Reason for exclusion: Ineligible medication**

Rasmussen DD, Alexander L, Malone J, Federoff D, Froehlich JC. The Î±2-adrenergic receptor agonist, clonidine, reduces alcohol drinking in alcohol-preferring (P) rats. Alcohol. 2014;48(6):543–9.

**Reason for exclusion: Ineligible medication**

Reid ML, Hubbell CL, Douglass AV, Boedeker KL, Reid LD. Research with rats germane to medication for alcoholism: consequences of noncompliance. Alcohol. 2001;24(3):169–77.

**Reason for exclusion: Ineligible study design**

Rukstalis MR, Stromberg MF, O’Brien CP, Volpicelli JR. 6-beta-naltrexol reduces alcohol consumption in rats. Alcohol Clin Exp Res. 2000;24(10):1593–6.

**Reason for exclusion: Ineligible medication**

Sandi C, Borrell J, Guaza C. Naloxone decreases ethanol consumption within a free choice paradigm in rats. Pharmacol Biochem Behav. 1988;29(1):39–43.

**Reason for exclusion: Ineligible medication**

Shoemaker WJ, Vavrousek-Jakuba E, Arons CD, Kwok FC. The acquisition and maintenance of voluntary ethanol drinking in the rat: effects of dopaminergic lesions and naloxone. Behav Brain Res. 2002;137(1–2):139–48.

**Reason for exclusion: Ineligible medication**

Spanagel R, Pendyala G, Abarca C, Zghoul T, Sanchis-Segura C, Magnone MC, et al. The clock gene Per2 influences the glutamatergic system and modulates alcohol consumption. Nat Med. 2005;11(1):35–42.

**Reason for exclusion: Ineligible study design**

Stasiak A, Fogel WA. High voluntary alcohol consumption, in experimental liver cirrhosis is hardly responsive to opioid antagonist treatment. J Physiol Pharmacol. 2008;59(1):101–14.

**Reason for exclusion: Ineligible study design**

Stromberg MF, Sengpiel T, Mackler SA, Volpicelli JR, O’Brien CP, Vogel WH. Effect of naltrexone on oral consumption of concurrently available ethanol and cocaine in the rat. Alcohol. 2002;28(3):169–79.

**Reason for exclusion: Ineligible study design**

Tabbara RI, Li Z, Fletcher PJ, LÃª AD. The serotonin 2C receptor agonist lorcaserin, alone and in combination with the opioid receptor antagonist naltrexone, attenuates binge-like ethanol drinking. Addict Biol. 2021;26(5):e13040.

**Reason for exclusion: Ineligible study design**

Taksande BG, Nambiar S, Patil S, Umekar MJ, Aglawe MM, Kotagale NR. Agmatine reverses ethanol consumption in rats: Evidences for an interaction with imidazoline receptors. Pharmacol Biochem Behav. 2019;186:172779.

**Reason for exclusion: Ineligible medication**

Tanchuck MA, Yoneyama N, Ford MM, Fretwell AM, Finn DA. Assessment of GABA-B, metabotropic glutamate, and opioid receptor involvement in an animal model of binge drinking. Alcohol. 2011;45(1):33–44.

**Reason for exclusion: Ineligible phenomena of interest**

Tezcan K, Yananli HR, Demirkapu MJ, GÃ¶ren MZ, Sakalli HE, Colombo G, et al. The effect of telmisartan, an angiotensin receptor blocker, on alcohol consumption and alcohol-induced dopamine release in the nucleus accumbens. Alcohol. 2021;96:73–81.

**Reason for exclusion: Ineligible medication**

Valenza M, Blasio A, DiLeo A, Cottone P, Sabino V. Sigma receptor-induced heavy drinking in rats: Modulation by the opioid receptor system. Pharmacol Biochem Behav. 2020;192:172914.

**Reason for exclusion: Ineligible study design**

Vallender EJ, RÃ¼edi-Bettschen D, Miller GM, Platt DM. A pharmacogenetic model of naltrexone-induced attenuation of alcohol consumption in rhesus monkeys. Drug Alcohol Depend. 2010;109(1–3):252–6.

**Reason for exclusion: Ineligible study design**

Vengeliene V, Leonardi-Essmann F, Sommer WH, Marston HM, Spanagel R. Glycine transporter-1 blockade leads to persistently reduced relapse-like alcohol drinking in rats. Biol Psychiatry. 2010;68(8):704–11.

**Reason for exclusion: Ineligible study design**

Viudez-MartÃ­nez A, GarcÃ­a-GutiÃ©rrez MS, Fraguas-SÃ¡nchez AI, Torres-SuÃ¡rez AI, Manzanares J. Effects of cannabidiol plus naltrexone on motivation and ethanol consumption. Br J Pharmacol. 2018;175(16):3369–78.

**Reason for exclusion: Ineligible study design**

Walker BM, Rasmussen DD, Raskind MA, Koob GF. alpha1-noradrenergic receptor antagonism blocks dependence-induced increases in responding for ethanol. Alcohol. 2008;42(2):91–7.

**Reason for exclusion: Ineligible study design**

Wegelius K, Honkanen A, Korpi ER. Benzodiazepine receptor ligands modulate ethanol drinking in alcohol-preferring rats. Eur J Pharmacol. 1994;263(1–2):141–7.

**Reason for exclusion: Ineligible medication**

Weiss F, Mitchiner M, Bloom FE, Koob GF. Free-choice responding for ethanol versus water in alcohol preferring (P) and unselected Wistar rats is differentially modified by naloxone, bromocriptine, and methysergide. Psychopharmacology (Berl). 1990;101(2):178–86.

**Reason for exclusion: Ineligible medication**

Williams KL, Broadbridge CL. Potency of naltrexone to reduce ethanol self-administration in rats is greater for subcutaneous versus intraperitoneal injection. Alcohol. 2009;43(2):119–26.

**Reason for exclusion: Ineligible comparator**

Williams KL, Kane EC, Woods JH. Interaction of morphine and naltrexone on oral ethanol self-administration in rhesus monkeys. Behav Pharmacol. 2001;12(5):325–33.

**Reason for exclusion: Ineligible study design**

Williams KL, Woods JH. Oral ethanol-reinforced responding in rhesus monkeys: effects of opioid antagonists selective for the mu-, kappa-, or delta-receptor. Alcohol Clin Exp Res. 1998;22(8):1634–9.

**Reason for exclusion: Ineligible study design**

Wilson AW, Neill JC, Costall B. An investigation into the effects of 5-HT agonists and receptor antagonists on ethanol self-administration in the rat. Alcohol. 1998;16(3):249–70.

**Reason for exclusion: Ineligible study design**

Yardley MM, Neely M, Huynh N, Asatryan L, Louie SG, Alkana RL, et al. Multiday administration of ivermectin is effective in reducing alcohol intake in mice at doses shown to be safe in humans. Neuroreport. 2014;25(13):1018–23.

**Reason for exclusion: Ineligible comparator**

Yardley MM, Wyatt L, Khoja S, Asatryan L, Ramaker MJ, Finn DA, et al. Ivermectin reduces alcohol intake and preference in mice. Neuropharmacology. 2012;63(2):190–201.

**Reason for exclusion: Ineligible comparator**

Zalewska-Kaszubska J, Bajer B, Gorska D, Andrzejczak D, Dyr W, BieÅ„kowski P. Voluntary alcohol consumption and plasma beta-endorphin levels in alcohol preferring rats chronically treated with lamotrigine. Physiol Behav. 2015;139:7–12.

**Reason for exclusion: Ineligible medication**

Zalewska-Kaszubska J, GÃ³rska D, Dyr W, Czarnecka E. Effect of chronic acamprosate treatment on voluntary alcohol intake and beta-endorphin plasma levels in rats selectively bred for high alcohol preference. Neurosci Lett. 2008;431(3):221–5.

**Reason for exclusion: Ineligible phenomena of interest**

Zhou Y, Crowley R, Prisinzano T, Kreek MJ. Effects of mesyl salvinorin B alone and in combination with naltrexone on alcohol deprivation effect in male and female mice. Neurosci Lett. 2018;673:19–23.

**Reason for exclusion: Ineligible medication**

Zhou Y, Kreek MJ. Clinically utilized kappa-opioid receptor agonist nalfurafine combined with low-dose naltrexone prevents alcohol relapse-like drinking in male and female mice. Brain Res. 2019;1724:146410.

**Reason for exclusion: Ineligible medication**

Zhou Y, Kreek MJ. Combination of Clinically Utilized Kappa-Opioid Receptor Agonist Nalfurafine With Low-Dose Naltrexone Reduces Excessive Alcohol Drinking in Male and Female Mice. Alcohol Clin Exp Res. 2019;43(6):1077–90.

**Reason for exclusion: Ineligible study design**

Zhou Y, Rubinstein M, Low MJ, Kreek MJ. V1b Receptor Antagonist SSR149415 and Naltrexone Synergistically Decrease Excessive Alcohol Drinking in Male and Female Mice. Alcohol Clin Exp Res. 2018;42(1):195–205.

**Reason for exclusion: Ineligible study design**
